# Effects of xylooligosaccharide supplementation on methane emissions, rumen microbiome, and serum metabolome in lactating Jersey cows

**DOI:** 10.1080/10495398.2026.2743468

**Published:** 2026-10-22

**Authors:** Xiaonan Zhou, Zhiyan Zhao, Lei Zhao, Qiyu Diao, Lifeng Dong, Xiaolong Kang

**Affiliations:** ^a^ College of Animal Science and Technology, Ningxia University, Key Laboratory of Ruminant Molecular and Cellular Breeding, Ningxia University, Yinchuan, China; ^b^ Yinchuan Animal Husbandry Technical Extension and Service Center, Yinchuan, China; ^c^ Feed Research Institute, Chinese Academy of Agricultural Sciences, Beijing, China

**Keywords:** xylooligosaccharides, methane, ruminal microbiota, metabolomics, Jersey cow

## Abstract

Xylooligosaccharides (XOS) are recognized as effective prebiotics that can mitigate methane (CH_4_) emissions. However, the specific relationship between the effects of XOS on CH_4_ emissions and alterations in the ruminal microbiota and their metabolites remains unclear. To investigate the relationship between XOS and CH_4_ emissions, 48 Jersey cows were randomly divided into two groups: the XOS group (group T) and the control group (group C). Group T received 25 g/d of XOS, while group C received 0 g/d of XOS added to the basal diet. Three randomly selected cows from each group, exhibiting consistent milk production and lactation stage, were analyzed for CH_4_ emissions detection, 16S rRNA sequencing, and untargeted metabolomics analysis. The addition of XOS significantly reduced CH_4_ emissions and increased both the number of observed species and the abundance-based coverage estimator (ACE) index of ruminal microbiota compared to group C. Sixteen differential metabolites were identified between the two groups; these differential metabolites were correlated with microbiota, including *Chloroflexi*, *Acidobacteriota*, and *Succiniclasticum.* The study suggests that XOS may regulate ruminant CH_4_ emissions by influencing the microbiota, thereby providing a rationale for CH_4_ control.

## Introduction

Global warming, a direct consequence of the greenhouse effect, is primarily driven by the increase in greenhouse gases and ozone layer depletion, both of which are linked to methane (CH_4_).^1^ Livestock contribute to 14.5% of anthropogenic greenhouse gas emissions, with CH_4_ emissions from ruminant intestines accounting for 5.8%.^2^ As global demand for milk and meat is projected to double by 2050, CH_4_ emissions from livestock are expected to increase significantly.^3^ Therefore, it is imperative to reduce CH_4_ emissions from animal husbandry, particularly from ruminants.

CH_4_ is produced in ruminants through the synergistic activity of diverse ruminal microbiota. The distinctive ruminal microbiota in ruminants facilitates the digestion of a variety of high-fiber feeds, during which CH_4_ is generated as a natural byproduct.^4^ In addition, carbon dioxide (CO_2_) and hydrogen (H_2_) generated by ruminal microbiota can be converted to CH_4_ by methanogenic bacteria, which use these gases as primary substrates.^5^ Different substrates utilized by methanogenic bacteria correspond to distinct CH₄ production pathways, with the H_2 _otrophic pathway, which catalyzes the conversion of CO_2_ to CH_4_, being dominant in the rumen.^6^ As a result, CH_4_ production convert a portion of the ruminant’s energy into CH_4_, accounting for approximately 2–12% of the total feeding energy.^7^ This process ultimately lowers the animal’s energy utilization efficiency.

The production of CH_4_ in anaerobic environments is primarily driven by methanogenic archaea, which metabolize substrates such as H_2_ and CO_2_ to generate CH_4_. Xylooligosaccharides (XOS), recognized as prebiotics, exhibit complex mechanistic interactions with CH_4_ production by modulating the composition and metabolic activity of gut microbiota. In broilers, dietary supplementation with XOS and arabinoxylan polysaccharides has been shown to improve growth efficiency by reducing intestinal epithelial cell turnover.^8^ Furthermore, a study conducted on piglets demonstrated that XOS supplementation promotes the growth of fiber-degrading gut microbiota in both suckling and weaned piglets.^9^ The short-chain fatty acids (SCFAs) produced through the fermentation of XOS reduce intestinal pH.^10^ Given that methanogenic archaea are relatively sensitive to pH fluctuations, an acidic environment may inhibit their proliferation and methanogenic activity.^11^ While these observations indicate plausible pathways, direct mechanistic evidence in ruminants remains limited.

To address this gap, our previous study demonstrated that XOS can increase milk production, energy, and feed utilization efficiency, and reduce gut CH_4_ emissions in Jersey cows.^12^ Nevertheless, it remains uncertain whether the impact of XOS on the performance of Jersey cows is mediated by its influence on the composition or abundance of the gut microbiota. Building upon this research, we conducted 16S rRNA sequencing and untargeted metabolomics analysis of rumen fluid and serum from Jersey cows in both the XOS and control groups. Our findings suggest that XOS inhibits CH_4_ emissions in Jersey cows, possibly by exerting effects on the ruminal microbiota and blood metabolite profiles. These findings elucidate the mechanism by which XOS reduces CH_4_ emissions in dairy cattle, thereby providing a theoretical foundation for the development of environmentally friendly feed additives for ruminants.

## Materials and methods

### Animals and experimental design

Detailed experimental procedures are described in our previous study.^12^ Briefly, 48 Jersey cows were randomly assigned to group C or group T to ensure baseline comparability, with no significant differences observed between the groups in age, days in milk (DIM), milk yield, and dry matter intake (DMI) (Table 1). Throughout the experiment, each cow was housed in an individual pen with a separate trough. The feed intake per cow was calculated by weighing and recording the feed offered and the leftovers at fixed times each day. The detailed method for measuring DMI per dairy cow is as described in our previous research.^12^ The basal diet was supplemented with XOS at 0 g/d for group C and 25 g/d for group T. The total duration of the experimental period was 60 days, which included an initial pre-feeding phase of 10 days, followed by a formal experimental phase lasting 50 days. The dietary composition and nutrient content of the feed ingredients were consistent with those reported in a previous study,^12^ as detailed in Table S1. During the test period, all experimental animals had free access to feed and water, under identical feeding management practices and environmental conditions. The total mixed ration (TMR) was uniformly distributed by the farm at 9:00 and 16:00 every day. The enclosure was equipped with an automated brick-licking system, an automatic cleaning device, and a daily maintenance regimen for the cows’ bedding and water troughs, ensuring a hygienic and comfortable environment. After the feeding period, three cows in group C were designated as C0661, C2562, and C0651, while those in group T were labeled as T2138, T0686, and T0747. 16S rRNA sequencing and untargeted metabolomics analysis were performed. The XOS used in the present study was provided by Yicong Bio-Tech Co., Ltd. (Zhengzhou, Henan, China) and was produced from steam-exploded corncobs^12^ this XOS product contained 35% XOS with 65% maltodextrin as the carrier.^13^ This animal experiment received approval from the Science and Technology Ethics Committee of Ningxia University (24-F-062).

**Table 1.** Days in milk, milk yield, CH_4_ yield, and DMI of each cow in the XOS and control groups.

Group	Experimental cow number	Age in months		Days in milk (d)		Milk yield (kg/d)	CH_4_ yield (g/d)		DMI (kg/d)
Control group	C0661	48.66	48.76 ± 0.17^a^	214.00	190 ± 23.50^a^	19.37 ± 2.55^a^	486.44 ± 16.70^a^		
	C2562	48.53		213.00				17.5 ± 1.71	
	C0651	49.09		143.00					
XOS group	T2138	49.06	48.69 ± 0.20^a^	206.00	185 ± 17.62^a^	18.60 ± 1.80^a^	379.58 ± 28.29^b^		
	T0686	48.6		199.00				17.9 ± 1.59	
	T0747	48.4		150.00					

Different lowercase superscript letters within the same column indicate a significant difference between group C and group T at *P* < 0.05; the same letter indicates no significant difference.

### Sample collection

Rumen fluid was collected from each cow using a steriliDifferent lowercase superscript letters within the same row indicate a significant difference between the control group (C) and the XOS group (T) at p < 0.05; the same letter indicates no significant difference.zed oral sampler and a 200 mL syringe before morning feeding on the final day of the experiment. The initial 200 mL of fluid collected was discarded to prevent contamination from saliva. The rumen fluid from each cow was filtered through four layers of gauze, transferred into cryotubes, and immediately frozen in liquid nitrogen before being stored at −80 °C for ruminal microbiota analysis. On the final day of the experiment, blood samples were collected from each cow. Blood was drawn from the median caudal vein using a vacuum blood collection tube. After a brief resting period, the blood samples were placed in a centrifuge, and the serum was separated by centrifuging the samples at a rotational speed of 3500 r/min for 20 min. The serum was then immediately frozen in liquid nitrogen and subsequently stored at −80 °C for untargeted metabolomics analysis.

### Measurement of CH_4_ yield

Enteric CH_4_ yield was quantified using the GreenFeed Large Animal System (C-Lock Inc., Rapid City, SD, USA).^14^ CH_4_ concentrations were measured automatically using non-dispersive infrared and paramagnetic sensors. Each cow was acclimated to the systems over a 10-day training period. The system, equipped with a radio-frequency identification reader, was configured to enforce a minimum access interval of 3 hours per cow. A valid measurement event was defined as a visit during which the animal’s head remained in the sampling hood for >3 min (mean visit duration in this study: 7 min 56 s). Shorter visits were automatically discarded by the system. Data were collected over 14 days (days 32–44 of the experiment) to ensure that a minimum of 20 valid measurements were obtained for each cow, which was necessary to generate a reliable estimate of average daily CH_4_ production. To ensure data accuracy, the gas sensors were calibrated weekly using a zero-gas (99.999% N_2_) and a span gas containing known concentrations of CH_4_. The specific measurements were those taken in the previous study.^14^

### Rumen fluid 16S rRNA sequencing

#### DNA extraction, PCR amplification, and sequencing

Total genomic DNA was extracted from rumen fluid samples using the DNeasy PowerSoil Kit (Qiagen, Hilden, Germany). The purity and concentration of the extracted DNA were assessed through agarose gel electrophoresis, and the samples were diluted to 1 ng/μL for polymerase chain reaction (PCR). The V4 region of the bacterial 16S rRNA gene was amplified using specific primers, 515 F and 806R (Table S2), which included a barcode. All PCR reactions were conducted using 15 µL of Phusion^®^ High-Fidelity PCR Master Mix (New England Biolabs). The thermal cycling conditions were as follows: initial denaturation at 98 °C for 1 min, denaturation at 98 °C for 10 s, annealing at 50 °C for 30 s, extension at 72 °C for 30 s, followed by a total of 30 cycles. PCR products were detected by 2% agarose gel electrophoresis. For library construction and quality control, the TruSeq^®^ DNA PCR-Free Sample Preparation Kit was utilized. Sequencing was performed using the Illumina NovaSeq 6000 once the library quality was confirmed to be satisfactory.

#### 16S rRNA sequencing data analysis

Raw sequencing reads were demultiplexed and trimmed to remove barcodes and primers, after which paired-end reads were merged using FLASH (version: 1.2.7) to generate raw tags. Subsequently, these raw tags were subjected to stringent quality control filtering^15^ to obtain clean tags for downstream analysis. Following the tag quality control workflow of QIIME (version: 1.9.1)^16^ as follows: (a) Tags interception: the Raw Tags were truncated from the first low-quality base site where the number of consecutive low-quality bases (with a default quality threshold of ≤19) reaches a set length (default length value is 3); (b) Tags length filtering: the Tags dataset obtained after Tags interception were further filtered to remove consecutive high-quality bases that are less than 75% of the tags length. Tags obtained after the above processing must be processed to remove chimeric sequences, utilizing sequences through VSEARCH (https://github.com/torognes/vsearch/),^17^ and a species annotation database for the detection of chimeric sequences, ultimately removing the chimeric sequences,^18^ to obtain the effective tags. The Uparse algorithm (Uparse v7.0.1001, http://www.drive5.com/uparse/)^19^ was utilized to cluster all effective tags from all samples, clustering the sequences into operational taxonomic units (OTUs) with an identity threshold of 97%. Species annotation was performed on the OTU sequences; the Mothur method was used to analyze the species annotation with the SSUrRNA database (with a threshold set as 0.8–1)^20^ of SILVA 138 (http://www.arb-silva.de/),^21^ to obtain taxonomic information and to count the samples at each taxonomic level separately regarding each sample’s microbiota composition. All OTUs were aligned by MUSCLE^22^ (version: 3.8.31, http://www.drive5.com/muscle/) to determine the phylogenetic relationships of all OTU representative sequences. Finally, the data from each sample were standardized, using the sample with the smallest data volume as the benchmark for this process. Subsequent analyses of α and β diversity were conducted based on the standardized data. Rarefaction curves, Rank abundance, and species accumulation curves were plotted using R software (version: 2.15.3). Statistical comparisons of α and β diversity indices between groups were calculated using QIIME software (version: 1.9.1) in R. To compare α-diversity indices between groups, data normality was first determined using the Shapiro-Wilk test. This was followed by the application of either the Student’s t-test (for parametric comparisons) or the Wilcoxon rank-sum test (for non-parametric comparisons). For β-diversity, differences in microbial community composition were statistically evaluated using permutational multivariate analysis of variance based on Bray-Curtis distances. Unweighted unifrac was calculated, and Unweighted Pair-group Method with Arithmetic Means (UPGMA) sample clustering trees were constructed using QIIME software (version: 1.9.1). Principal component analysis (PCA) and non-metric multidimensional scaling (NMDS) plots were plotted using R software (version: 2.15.3). The PCA analysis employed the ade4 and ggplot2 packages (version: 3.4.4), while the NMDS analysis utilized the vegan package within R software (version: 2.15.3). t-test analysis was employed to identify species with significant differences between groups ( *P* < 0.05). Clustering heatmaps were generated using the pheatmap package in R (version: 2.15.3) to normalize the species/OTU abundance data through z-score transformation, and *P* < 0.05 was deemed statistically significant. To quantify the contribution of individual species to the observed differences between groups, Simper (Similarity percentage) analysis^23^,^24^ was conducted via the simper function of the vegan package in R software (version: 2.15.3). To identify biomarkers with statistically significant differences between groups, we performed LEfSe (linear discriminant analysis effect size) analysis using the default parameters and an LDA score filtering threshold of 4.0. Metastats analysis was employed in R software (version: 2.15.3) to perform permutation tests between groups at each classification level to obtain *P* values. Subsequently, the Benjamini and Hochberg False Discovery Rate method was applied to correct the *P* values and derive *q* values.^25^

### Untargeted metabolomic sequencing

#### LC-MS/MS-based untargeted metabolomics

A total of 100 μL of serum sample was transferred to an EP tube, followed by the addition of 400 μL of 80% methanol in water. The mixture was vortexed and then shaken, followed by a 5 min incubation in an ice bath. Subsequently, the sample was centrifuged at 15,000 g and 4 °C for 20 min. A specific volume of the supernatant was collected and diluted with mass spectrometry-grade water to achieve a final concentration of 53% methanol. The sample was then centrifuged again at 15,000 g and 4 °C for 20 min, and the supernatant was collected for analysis by liquid chromatography-tandem mass spectrometry (LC-MS/MS).^26^ UHPLC-MS/MS analyses were conducted using a Vanquish UHPLC system (Thermo Fisher, Germany) coupled with an Orbitrap Q Exactive^™^ HF mass spectrometer (Thermo Fisher, Germany) at Novogene Co., Ltd. (Beijing, China). The downstream data (.raw) files were imported into Compound Discoverer 3.1 (CD3.1, Thermo Fisher) to perform peak alignment, peak picking, and quantitation for each metabolite. The main parameters were set as follows: retention time tolerance, 0.2 minutes; actual mass tolerance, 5 ppm; signal intensity tolerance, 30%; signal/noise ratio, 3; and minimum intensity. After that, peak intensities were normalized to the total spectral intensity. The normalized data were used to predict the molecular formula based on additive ions, molecular ion peaks, and fragment ions. Then peaks were matched with the mzCloud (https://www.mzcloud.org/), mzVault, and Masslist databases to obtain the accurate qualitative and relative quantitative results. Statistical analyses were conducted using the statistical software R (version: 2.15.3), Python (version: 2.7.6), and CentOS (version: 6.6). When the data were not normally distributed, normal transformations were attempted using the area normalization method.

#### Analysis of untargeted metabolomics sequencing data

In the multivariate statistical analysis section, PCA and Partial least squares discriminant analysis (PLS-DA) were performed using the metaX R package.^27^ In the univariate analysis section, statistical significance between the two groups was calculated for each metabolite using t-tests, and *P* < 0.05 was deemed statistically significant. Additionally, the fold change (FC) value was used to represent the relative difference in metabolite levels between the two groups. The metabolites with VIP > 1.0, FC > 1.2, or FC < 0.833 and *P* < 0.05^28–30^ were considered to be differential metabolites. The volcano plot was employed to filter metabolites of interest, based on log_2_ (fold change) and -log_10_(P-value) of metabolites, utilizing the R package ggplot2 (version: 3.4.4). Correlation analysis using the Pearson correlation coefficient was conducted among different metabolites with the R function cor(). Statistical significance was assessed through cor.mtest() in R. Correlation maps were visualized using the corrplot package in R. Additionally, bubble plots were generated using the ggplot2 package in R (version: 3.4.4). These metabolites were annotated using the Kyoto Encyclopedia of Genes and Genomes (KEGG) database (https://www.genome.jp/kegg/pathway.html) and the Human Metabolome Database (HMDB) (https://hmdb.ca/metabolites), when *P* < 0.05 of metabolic pathway, metabolic pathway was considered as statistically significant enrichment.

### Analysis of differential metabolite-microbe interactions in the bovine rumen

To investigate the association between bovine ruminal microbiota and differential metabolites following the addition of XOS, 16 differential metabolites were correlated with the top 10 microbiota abundance data at both the phylum and genus levels. Spearman correlations are more effective than other methods (e.g., Pearson correlation) in determining gene expression and the relative abundance of microbiomes. Spearman rank correlation coefficients and corresponding *P* values were calculated using the cor.test() function, while the p.adjust(P, method = "BH") function in R (version 2.15.3) was employed to perform multiple comparison corrections of the *P* value, yielding the FDR value. The correlations among differential metabolites were visualized using the R package corrplot (version: 0.92), and significantly correlated microbial and differential metabolites were illustrated using Cytoscape (version: 3.10.1).

## Results

### Xylooligosaccharides reduce methane emissions

CH_4_ emissions for the two groups (C/T) were quantified, and the results are presented in Table 1. Our findings showed that CH_4_ emissions for group C were 486.44 ± 16.70 g/d, while those for group T were 379.58 ± 28.29 g/d (*P* < 0.05). Consistent with the results of our previous investigation, XOS significantly reduced CH_4_ emissions.^12^

### Xylooligosaccharides enhance the diversity of ruminal microbiota

A total of 497,738 reads were generated by 16S rRNA sequencing, with an average of 82,956 ± 2,346.22 reads (mean ± standard error of the mean [SEM]) obtained for each sample. The downstream data obtained from sequencing (Raw PE) were merged and subjected to quality control, resulting in a total of 491,689 clean tags. Subsequently, chimera filtering was performed to obtain effective tags, which were used for downstream analysis (Table S3). The rarefaction curve gradually flattened with increasing sequencing depth, indicating that the amount of sequencing data was progressively adequate and that the sequencing depth was sufficient to reflect the diversity of microbiota in the current samples (Fig. 1A). The rank clustering curves (Fig. 1B) demonstrated that, in the horizontal direction, group T spanned more extensively compared to group C, indicating that the species richness of microbiota in the rumen of cows in group T was higher. The results of the α-diversity analysis demonstrated that the number of observed species was significantly higher in group T than in group C (*P* = 0.026). Furthermore, both Chao1 and ACE indices were significantly elevated in group T compared to group C (*P* = 0.025 and *P* = 0.026, respectively). However, the differences between the two groups in Shannon and Simpson indices were not significant (*P* > 0.05) (Table 2). These results indicated that feeding XOS influenced the microbial composition of the rumen in Jersey cows. Additionally, the abundance of ruminal microbiota was observed to be elevated in dairy cows, although the diversity of ruminal microbiota remained unchanged.

**Figure 1.**
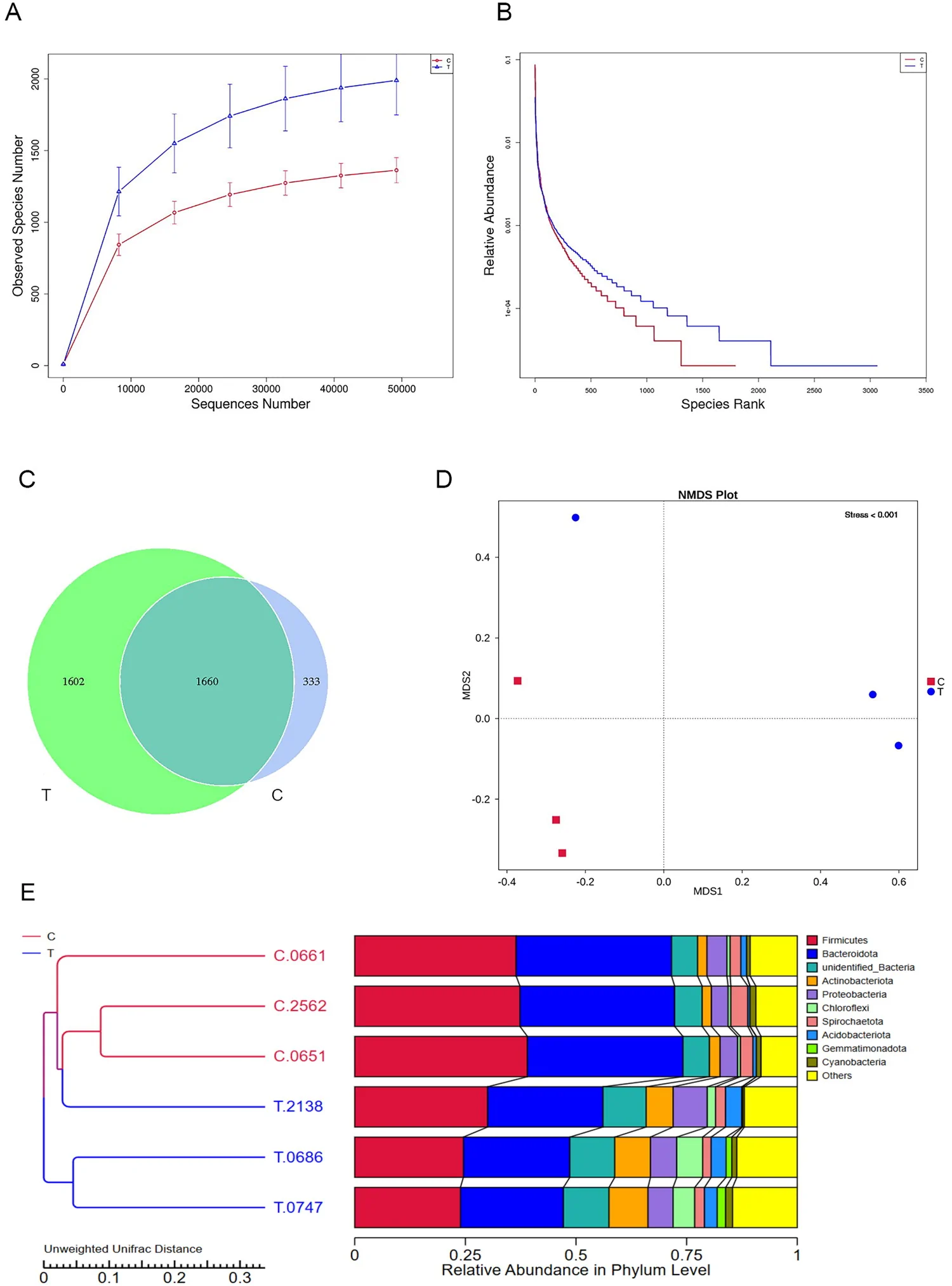
Analysis of OTUs from rumen fluid 16S rRNA gene sequencing. (A). Rarefaction curve. (B). Rank abundance curve. (C). Venn diagram. (D). NMDS plot. Each point represents a sample, and the distance between points reflects the degree of β-diversity. Samples from the same group are colored identically. The NMDS accurately reflects variations between samples when stress is less than 0.2. (E). UPGMA clustering tree based on unweighted UniFrac. *Note:* the left panel shows the UPGMA tree topology, and the right panel displays the relative abundance of taxa at the phylum level for each sample.

**Table 2.** Ruminal microbial α-diversity indices for dairy cows in the group T and group C.

Items	Group C	Group T	*P* value
Observed_species	1363 ± 107.68^b^	1991 ± 296.55^a^	0.026
Shannon’s diversity index	7.37 ± 0.56	8.42 ± 0.65	0.102
Simpson’s index	0.97 ± 0.015	0.99 ± 0.004	0.103
Chao1’s index	1463 ± 104.00^b^	2096.89 ± 298.44^a^	0.025
ACE’s index	1468.59 ± 107.71^b^	2120.55 ± 310.27^a^	0.026
Goods_coverage’s index	0.996	0.995	0.158
PD_whole_tree’s index	149.10 ± 19.06	197.13 ± 33.75	0.098

*Note:* Observed species: the number of species observed directly. Shannon: the total number of taxa in the sample and their proportion, the higher the community diversity and the more evenly distributed the species, the greater the Shannon index. Simpson characterizes the diversity and evenness of species distribution within the community. Chao1: estimates the total number of species contained in the community sample. ACE: estimated number of OTUs in the community. Goods coverage: index of sequencing depth. PD_whole_tree: affinities of species within the community. Data are presented as mean ± standard deviation. *P* values were calculated using the Student’s t-test. Different superscript letters within a row indicate a significant difference (*P* < 0.05).

The results of OTUs obtained from clustering indicated that the number of unique OTUs in group T is 4.8 times greater than that in group C. This suggests a significant difference in the composition of bacteria present in the rumen of group T (Fig. 1C). Additionally, the results of NMDS revealed that the composition of the ruminal microbiota differed between the two groups (Fig. 1D), indicating that the addition of XOS had altered the ruminal microbiota of the cows (stress < 0.001). A UPGMA phylogenetic tree was constructed based on the unweighted phylogenetic distance, visually representing the clustering structure formed by the microbial community composition. The results demonstrated that samples within each group clustered closely, with a clear differentiation between the two groups (Fig. 1E).

### Xylooligosaccharides alter the structure and composition of ruminal microbiota

A total of 3,598 OTUs were identified through a comparison with the SILVA 138 database for species annotation and the enumeration of distinct taxonomic levels. Of these, 3,554 OTUs (98.78%) were accurately annotated in the database. The percentage of annotations at the Kingdom level was 98.78%, at the Phylum level 89.52%, at the Class level 88.08%, at the Order level 82.05%, at the Family level 70.54%, and at the Genus level 46.30%. The taxonomic annotation of the microbiota of both groups revealed that at the phylum level, the predominant phyla included *Bacteroidota, Firmicutes*, and *Proteobacteria*, accounting for 47.43%, 31.34%, and 10.03% of the microbiota in group C, 44.87%, 30.80%, and 30.80% of the microbiota in group T, respectively (Fig. 2A). At the order level, the dominant microbiota were *Bacteroidales, Aeromonadales,* and *Veillonellales-Selenomonadales*, which accounted for 47.15%, 9.26%, and 5.7% in group C, 44.42%, 1.05%, and 2.30% in group T, respectively. At the family level, the dominant microbiota were *Prevotellaceae, Succinivibrionaceae*, and *Lachnospiraceae*, which accounted for 38.32%, 9.26%, and 7.54% in group C, 32.98%, 1.05%, and 8.07% in group T, respectively. At the genus level, the dominant microbiota were *Prevotella, Succinivibrionaceae_UCG-002*, and *Selenomonas*, which accounted for 35.36%, 8.92%, and 5.36% in group C, 29.86%, 0.87%, and 2.54% in group T, respectively (Fig. 2B).

**Figure 2.**
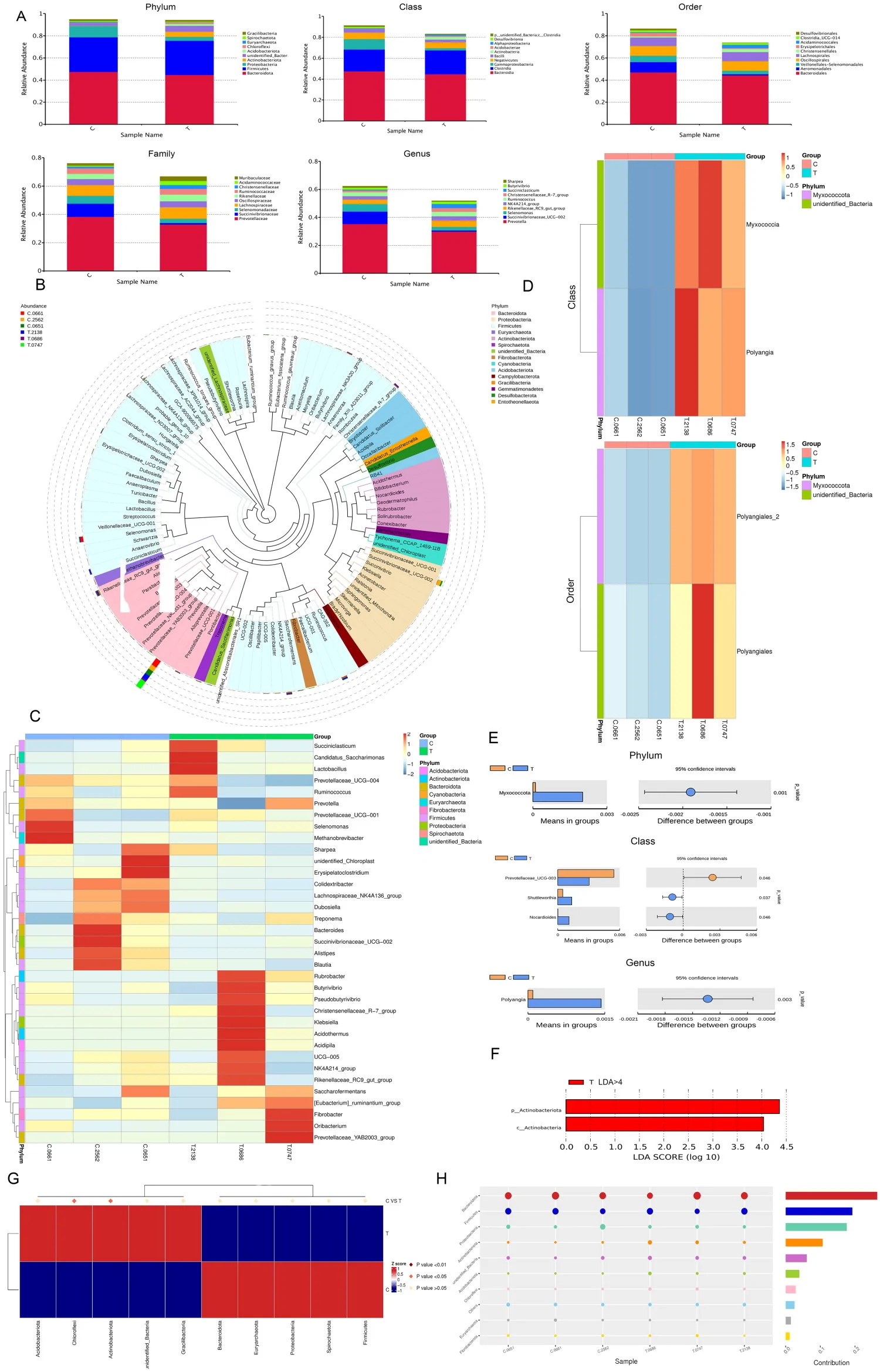
Analysis of the ruminal microbiota community based on 16S rRNA gene sequencing. (A). Relative abundance of the top 10 taxa at each taxonomic level. (B). Phylogenetic tree of species at the genus level. Note: the tree was constructed using representative sequences at the genus level. (C). Heatmap of abundance clustering at the genus level. (D). MetaStat analysis showing taxa with significant differences between groups. (E). t-test intergroup species difference analysis plot. (F). Bar plot of LDA effect sizes. Note: Taxa with an LDA score > 4.0 are considered discriminative biomarkers. (G). Heatmap of taxa with significant differences identified by MetaStat analysis. (H). Simper (similarity percentage) analysis showing the top 10 taxa contributing to dissimilarity between groups.

The clustering heatmap revealed that at the genus level, the abundance of *Prevotella* was significantly higher in group C than in group T (Fig. 2C). At the species level, intergroup differences indicated that *Myxococcia* and *Polyangia* were significant. In contrast, at the order level, *Polyangiales_2* and *Polyangiales* were identified as differential species (Fig. 2D). A t-test showed that the average abundance of the *Myxococcota* phylum was 0.0% in group C and 0.2% in group T, with a significant difference (*P* = 0.001) between the two groups. At the class level, the differing clusters included *Polyangia* (*P* = 0.003). At the genus level, the differing clusters were *Prevotellaceae UCG-003* (*P* = 0.046), *Shuttleworthia* (*P* = 0.037), and *Nocardioides* (*P* = 0.046) (Fig. 2E). This implies that these microbes exert a dominant influence on the XOS-mediated reduction of rumen CH_4_ emissions. LEfSe analysis identified two biomarkers with LDA scores greater than 4, namely Actinobacteria and *Actinobacteriota* (Fig. 2F). The presence of varying levels of taxonomic features indicated that the bovine ruminal microbiota possessed a broad and diverse profile. MetaStat analysis demonstrated that *Chloroflexi* and *Actinobacteriota* were significantly more abundant in group T than in group C at the phylum level (Fig. 2G). Simper analyses revealed that *Bacteroidota*, *Firmicutes*, and *Proteobacteria* contributed most to the differences between the two groups (Fig. 2H). This result delineated a distinct microbial profile associated with low CH_4_ emissions. Thus, these results imply that targeted modulation of the ruminal microbiota could be a viable strategy for CH_4_ mitigation.

### Functional enrichment analysis of ruminal microbiota

To assess the effect of XOS on microbial function in the rumen, we used sequences from the SILVA ribosomal RNA database (clustered from OTUs) as reference sequences for the Tax4Fun analysis. The results showed that the ruminal microbiota were involved in six major KEGG categories: cellular processes, environmental information processing, genetic information processing, human diseases, metabolism, and biological systems, with the metabolic pathway representing the largest proportion (Fig. 3A). Cluster heatmap analysis revealed that metabolism and environmental information processing were more abundant in group T (Fig. 3B). Notably, carbohydrate metabolism, amino acid metabolism, and lipid metabolism were significantly more abundant in group T compared to group C. Conversely, pathways related to endocrine and metabolic diseases, glycan biosynthesis, and metabolism were significantly less abundant in group T than in group C (Fig. 3C). t-test analysis demonstrated that starch and sucrose metabolism pathways exhibited significantly different abundances between the two groups, with a notably lower abundance in group T than in group C (*P* < 0.05) (Fig. 3D).

**Figure 3.**
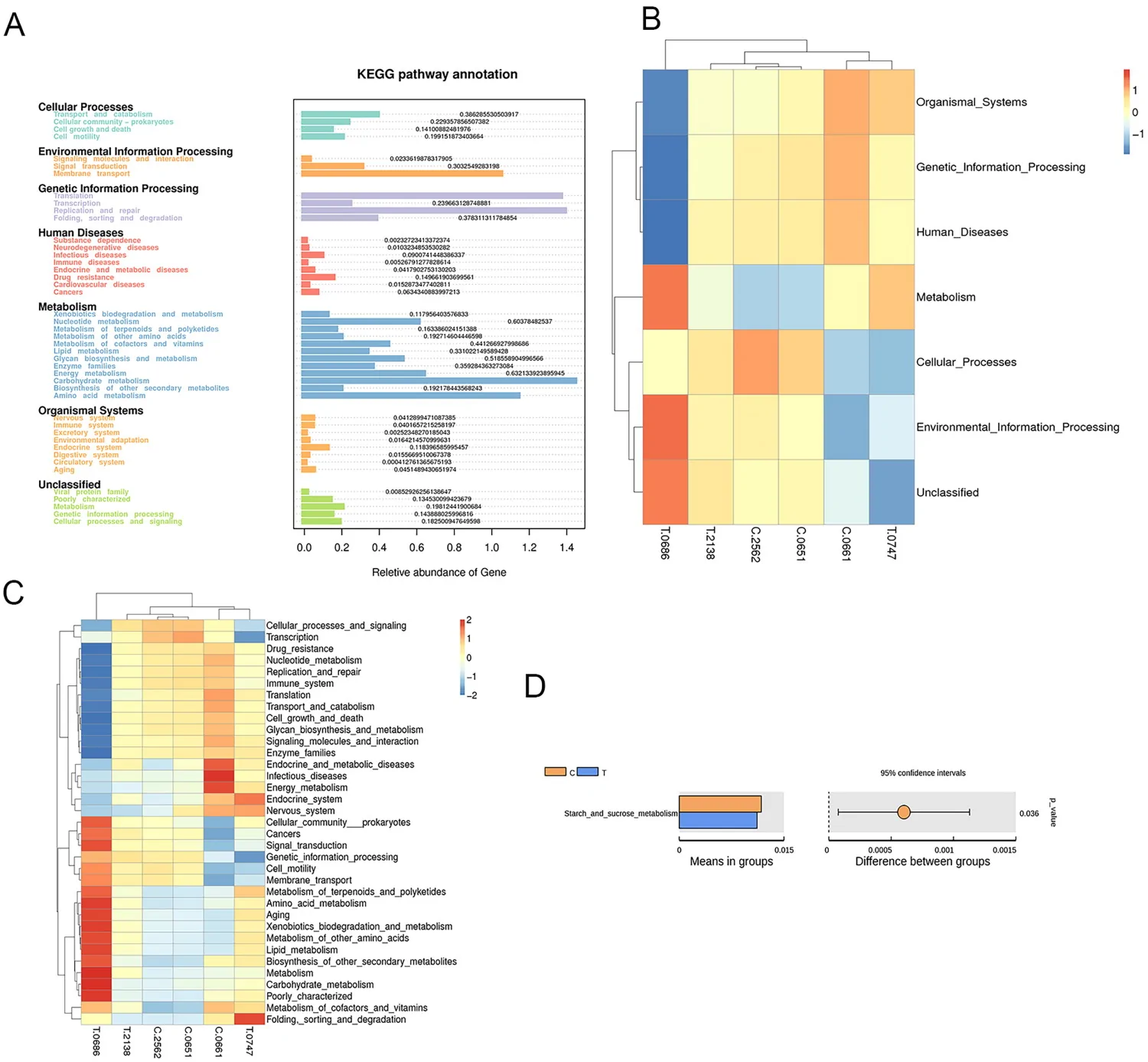
Functional annotation from 16S rRNA gene sequencing data. (A). KEGG pathway annotations. (B, C) Clustering heatmaps of predicted functional profiles at (B) KEGG level 1 and (C) KEGG level 2. (D). t-test analysis of differentially abundant pathways between groups.

### Xylooligosaccharides modulate various metabolic processes in bovine serum

To investigate the effect of XOS on metabolism, an untargeted metabolomics study was conducted using bovine serum samples from groups T and C, employing the LC-MS/MS technique.^31^ PCA indicated a clear separation between the group C and T samples (Fig. 4A). Furthermore, the results of 200 permutation tests of PLS-DA revealed that the intercept of the *Q*^2^ regression line with the Y-axis was less than 0 and *R*^2^ > *Q*^2^, suggesting that the model was not overfit and was highly reliable (Fig. S1A). The Pearson correlation coefficient among the quality control (QC) samples was calculated based on the relative quantitative values of the metabolites,^32^ with the *R*^2^ > 0.9, indicating excellent data reproducibility (Fig. 4B). The results of the differential metabolite receiver operating characteristic (ROC) curve showed that the area under the curve (AUC) was equal to 1. The AUC value is critical for assessing the sensitivity and specificity of the biomarker in prediction (Fig. S1B). LC-MS/MS untargeted metabolomics revealed differences in host metabolites between the two groups, identifying a total of 371 metabolites (Table S4), which were categorized into nine classes according to the HMDB annotations: 1 alkaloids and derivatives, 21 benzenoids, 73 lipids and lipid-like molecules, 10 nucleosides, nucleotides, and analogues, 44 organic acids and derivatives, 1 organic nitrogen compounds, 6 organic oxygen compounds, 17 organ heterocyclic compounds, and 5 phenylacetone and phenylacetone compounds (Table S5).

**Figure 4.**
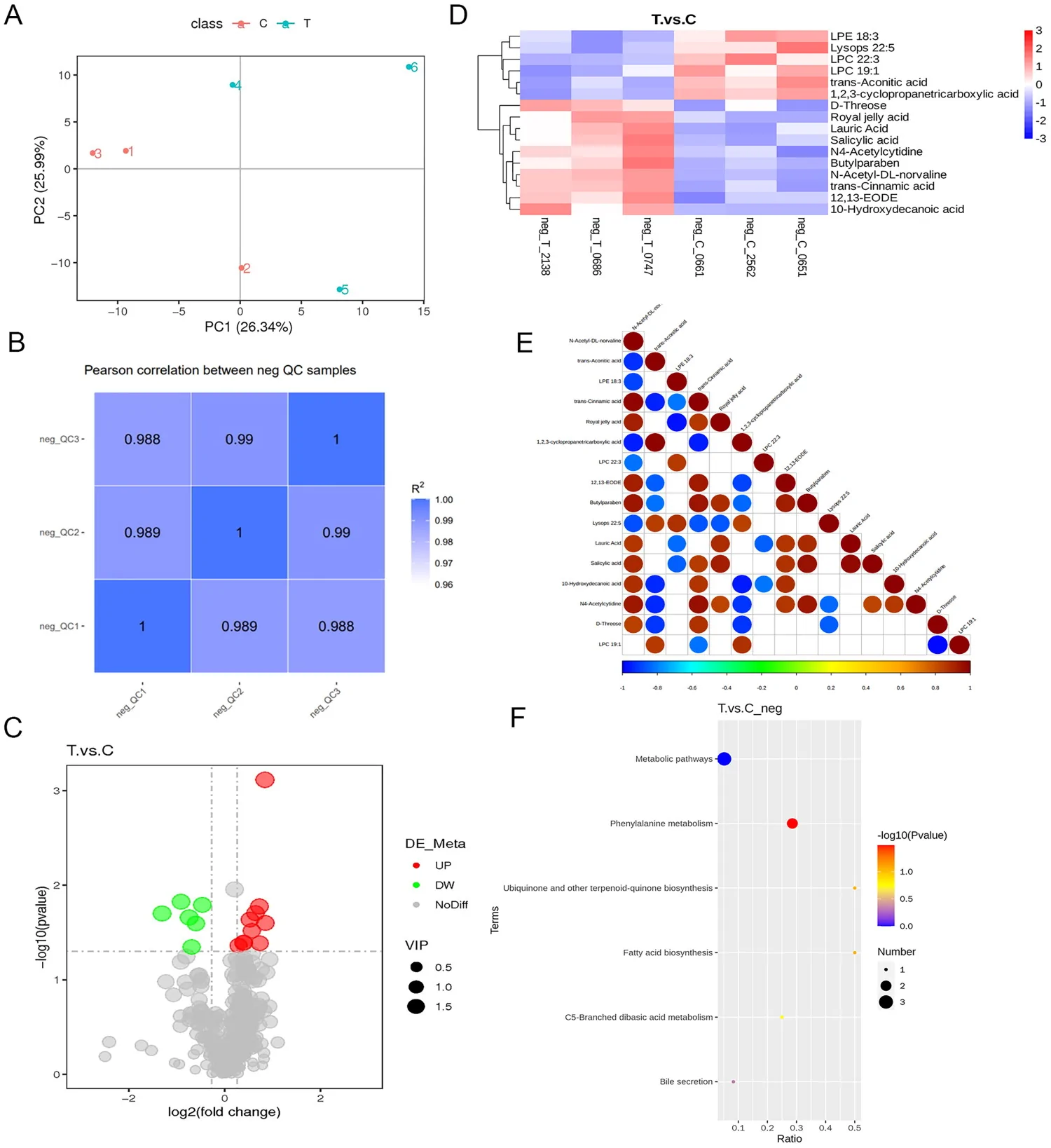
Serum untargeted metabolomic analysis. (A). PCA score plot. (B). Correlation heatmap of QC samples (all *R*^2^ > 0.9). (C). Differential metabolite volcano plot. (D). Differential metabolite clustering heatmap. (E). Differential metabolite correlation plot, note: Correlation is highest at 1 for a complete positive correlation (red), the lowest correlation is −1, which is a complete negative correlation (blue), and the part without color indicates *P* > 0.05. (F). KEGG pathway enrichment bubble plot.

The criteria for differential metabolite screening were set at VIP >1, *P* < 0.05, and FC ≥2, resulting in the identification of a total of 16 differential metabolites (Fig. 4C). Among these, 10 metabolites were up-regulated, and 6 metabolites were down-regulated in group T compared to group C (Table 3). The up-regulated metabolites in group T compared to group C included N-acetyl-DL-norvaline, trans-cinnamic acid, royal jelly acid, butylparaben, lauric acid, salicylic acid, 10-hydroxydecanoic acid, N4-acetylcytidine, 12,13-EODE,and D-threose. Notably, lauric acid and salicylic acid have been reported to be closely associated with CH_4_ production in the bovine gut.^33–35^ In addition, the levels of trans-aconitic acid, 1,2,3-cyclopropanetricarboxylic acid, and Lysops 22:5 were observed to be reduced in group T compared to group C (Fig. 4D). Significant correlations were noted among different metabolites, with butylparaben exhibiting a notably negative correlation with trans-aconitic acid (*P* < 0.05) and a positive correlation with trans-cinnamic acid and royal jelly acid (*P* < 0.05) (Fig. 4E). Using the KEGG database, we functionally annotated the 16 identified differential metabolites, revealing enrichment in several metabolic pathways, including phenylalanine metabolism, ubiquinone and other terpenoid-quinone biosynthesis, fatty acid biosynthesis, C5-branched dibasic acid metabolism, and bile secretion pathways (Fig. 4F).

**Table 3.** Fold changes of differential serum between the XOS group (group T) and the control group (group C).

Name	RT [min]	FC	*P* value	Up/down
N-Acetyl-DL-norvaline	4.96	1.79	0.00	up
Trans-Aconitic acid	2.05	0.53	0.01	down
LPE 18:3	8.64	0.73	0.02	down
Trans-Cinnamic acid	4.92	1.66	0.02	up
Royal jelly acid	6.85	1.57	0.02	up
1,2,3-cyclopropanetricarboxylic acid	2.27	0.41	0.02	down
LPC 22:3	10.14	0.60	0.02	down
12,13-EODE	7.64	1.44	0.02	up
Butylparaben	6.02	1.81	0.03	up
Lysops 22:5	9.35	0.66	0.03	down
Lauric Acid	8.71	1.48	0.03	up
Salicylic acid	6.14	1.32	0.04	up
10-Hydroxydecanoic acid	7.17	1.32	0.04	up
N4-Acetylcytidine	4.87	1.67	0.04	up
D-Threose	1.31	1.23	0.04	up
LPC 19:1	10.04	0.62	0.04	down

*Note:* RT = retention time. FC = fold change, calculated as the mean peak intensity in group T divided by the mean peak intensity in group C. FC > 1 indicates that the metabolite level was higher in group T, and FC < 1 indicates that it was lower in group T.

### Correlation analysis of differential metabolites with ruminal microbiota

The results of the correlation analysis between the differential metabolites and the ruminal microbiota indicated that, at the phylum level, *Actinobacteriota* and *unidentified_Bacteria* exhibited a positive correlation with N-acetyl-DL-norvaline, trans-cinnamic acid, and butylparaben, while demonstrating a negative correlation with LPC 19:1(Fig. 5A). *Acidobacteriota* showed a negative correlation with LPE 18:3 and LPC 22:3, but a positive correlation with royal jelly acid. *Chloroflexi* was positively correlated with royal jelly acid, 12,13-EODE, butylparaben, lauric acid, and salicylic acid, while negatively correlated with Lysops 22:5. *Euryarchaeota* displayed a positive correlation with trans-aconitic acid and 1,2,3-cyclopropanetricarboxylic acid, as well as with 12,13-EODE and butylparaben, whereas N4-acetylcytidine was negatively correlated (Fig. 5A). At the genus level, *Succinivibrionaceae_UCG-002* was positively correlated with LPE 18:3 but negatively correlated with royal jelly acid. Conversely, *Succiniclasticum* exhibited negative correlations with LPC 22:3 and LPC 19:1 (Fig. 5B). Notably, lauric acid has demonstrated significant inhibitory effects on CH_4_ emissions both *in vivo and in vitro*.[36,37] Lauric acid was up-regulated in group T of the current study, suggesting that the addition of XOS may regulate metabolites associated with CH_4_ metabolism, thereby influencing CH_4_ emissions in animals.

**Figure 5.**
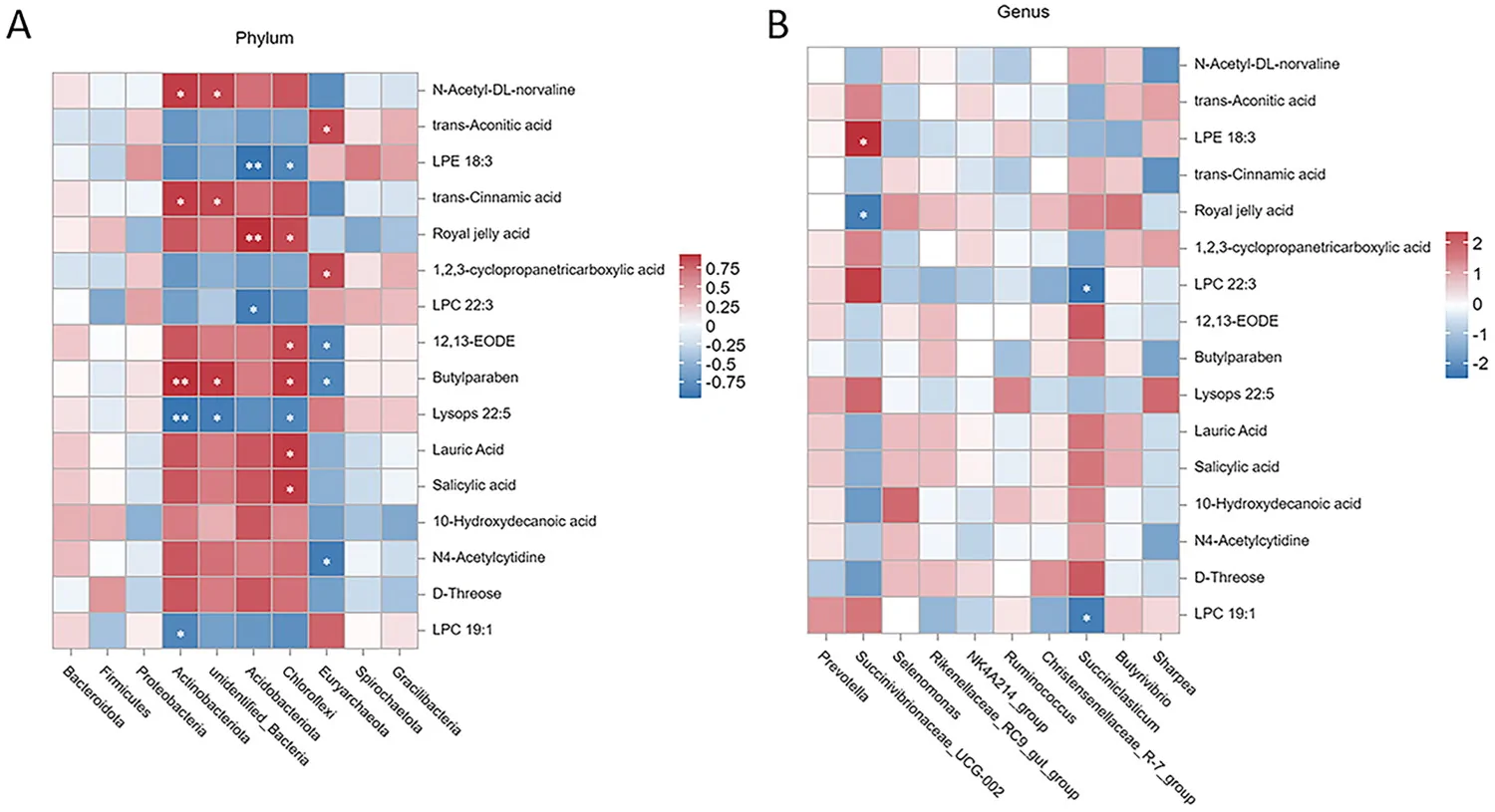
Correlation analysis between differential metabolites and the dominant bacterial phyla and genera. *Note:* the color intensity within each cell represents the Spearman correlation coefficient, with red indicating a positive correlation and blue indicating a negative correlation. “**” indicates *P* < 0.01, “*” indicates *P* < 0.05).

## Discussion

Rumen CH_4_ emissions from livestock are a significant concern for the livestock industry. Studies have reported that CH_4_ produced in the rumen of dairy cows accounts for 2.7% - 9.8% of total energy loss,^22^ and feed digestibility is lower in low-CH_4_ animals compared to high-CH_4_ animals.^38^ Consequently, CH_4_ emissions are one of the factors that reduce the production efficiency of the agricultural industry. XOS plays a crucial role in shaping the gut microbiota and regulating microbial composition, metabolite production, and substrate degradation rates.^39^ Therefore, the present study aimed to investigate the role of XOS supplementation in CH_4_ emissions in cattle.

We found that XOS increased the abundance of ruminal microbiota in dairy cows without altering its diversity. The effect of XOS on gut microbial diversity appears to be species - and protocol - dependent. While a 1% XOS diet reduced microbial diversity in chickens over 70 days,^40^ supplementation at 500 mg/kg had no significant effect on α-diversity in piglets after 28 days,^41^ and 100 g/t improved gut morphology without altering microbial abundance in piglets over 56 days.^42^ In contrast, our study in cattle using 25 g/d per cow for 60 days did not significantly alter ruminal microbial diversity. These divergent outcomes may be attributed to key differences in host species, supplementation dosage and form (e.g., percentage of diet vs absolute daily intake), and duration of administration, highlighting the context-specific nature of prebiotic effects. Although, *Prevotella* was the most abundant genus in both groups (∼30% of total sequences), and its overall abundance did not differ significantly. Additionally, it has been reported that the reduction of CH_4_ emissions is not exclusively associated with *Prevotella*.^43^^,^^44^ KEGG-based functional prediction indicated that the ruminal microbiota were actively engaged in core metabolic pathways, including carbohydrate, amino acid, and energy metabolism. These pathways are integral to rumen fermentation and have been consistently linked to the regulation of methanogenesis in ruminants.^45–48^ This demonstrates the potential of XOS in reducing CH_4_ emissions from ruminants.

The metabolic activities of ruminal microbiota may directly or indirectly affect various components of serum, including lipids, cholesterol, sugars, and other small-molecule metabolites.^49^,^50^ In this study, metabolomic analysis identified 16 differential metabolites, with lauric acid significantly elevated in group T relative to group C. The medium-chain fatty acid lauric acid has been shown to inhibit ruminal methanogenesis by up to 89% *in vitro* and 76% *in vivo*.^51^ Furthermore, lauric acid has been identified as a CH_4_ inhibitor,^35^ which may contribute to the reduced CH_4_ emissions observed in group T in this study. The differential metabolites identified were enriched in metabolic pathways, including phenylalanine metabolism and fatty acid biosynthesis. The fatty acid biosynthesis pathway plays a key role in rumen CH_4_ emissions, closely linked to shifts in volatile fatty acid (VFA) profiles. Inhibiting methanogenesis elevates ruminal H_2_ concentration, which in turn suppresses further H_2_ production and redirects H_2_ toward alternative metabolic sinks, including enhanced propionate and butyrate formation.^52^ Moreover, H_2_ can be utilized by rumen microbes for synthesizing microbial protein, thereby improving nitrogen utilization by the host.^53^ Thus, redirecting H_2_ away from methanogenesis toward VFA and microbial protein synthesis offers a dual benefit: reducing enteric CH_4_ emissions while enhancing energy and protein efficiency in ruminants. Our findings indicate that the abundance of ruminal microbiota is strongly correlated with serum metabolic activities. For instance, *Actinobacteriota* was positively correlated with royal jelly acid, but negatively correlated with N-Acetyl-DL-norvaline, trans-cinnamic acid, and butylparaben. Additionally, *Actinobacteriota* may be involved in amino acids metabolism, yielding amino acids and small peptides that can be absorbed by the host, thereby influencing serum levels of amino acids.^54^ IOverall, the ruminal *Actinobacteriota* interacts in a complex manner with serum metabolites. The present study also demonstrated that the addition of XOS led to a significant increase in lauric acid abundance, which has been shown to effectively inhibit CH_4_ emissions in the bovine gut.^37^ This finding aligns with the results of our study, suggesting that XOS may regulate CH_4_ emissions in animals by modulating metabolites associated with CH_4_ metabolism. However, the interactions and correlations between ruminal microbiota and metabolomics remain to be fully elucidated, necessitating further investigation.

While 16S rRNA sequencing provides reliable genus-level taxonomic profiling, its resolution at the species level is constrained by inherent methodological biases.^55^ Our findings indicate that XOS supplementation altered the abundance and composition of the ruminal microbial community. However, this study was conducted with a limited cohort of six Jersey cows, which may not fully represent broader dairy populations or production systems. Therefore, the generalizability of the results requires validation in larger, multi-breed trials. Furthermore, the observed correlations do not establish causality, and the long-term efficacy of XOS in mitigating CH_4_ emissions remains to be determined. Future studies should employ expanded cohorts, incorporate functional metagenomics to elucidate mechanistic pathways, and evaluate the sustainability of XOS effects over extended periods.

## Conclusion

The incorporation of 25 g/d of XOS into the diet resulted in a significant reduction in CH_4_ emissions from the rumen of cattle. Furthermore, enhanced the levels of metabolites such as lauric acid, trans-cinnamic acid, and N4-acetylcytidine, while simultaneously reducing the concentrations of trans-aconitic acid, 1,2,3-cyclopropanetricarboxylic acid, and Lysops 22:5. The finding of this study suggests that the reduction of CH_4_ emissions induced by XOS may be attributed to the interaction between ruminal microbiota and metabolites, which in turn influence microbial abundance within the rumen.

## Supplementary Material

supplementary tables S1_S5.docx

Supplementary figures.docx

## Data Availability

The 16S rRNA sequencing data have been uploaded to the NCBI database (https://dataview.ncbi.nlm.nih.gov/object/PRJNA1297622?reviewer=81rg27s1m2n2j0p0ae6bfpe6d, accessed on 28 Jul 2025). The untargeted metabolomic sequencing of this study is available from the corresponding author, [L.F. Dong, X.L. Kang], upon reasonable request.
