# Supplementary material for: Effects of xylooligosaccharide supplementation on methane emissions, rumen microbiome, and serum metabolome in lactating Jersey cows
Source: Anim Biotechnol. 2026 Oct 22;38(1):2743468. doi: 10.1080/10495398.2026.2743468 (PMC13715329; doi:10.1080/10495398.2026.2743468)
Supplement: Supplementary file 1 [file LABT_A_2743468_SM3771.docx]

**Regulatory effects of Xylooligosaccharides on methane production, rumen microbiome and serum metabolism in lactating Jersey cow**

*Xiaonan Zhou^a†^，Zhiyan Zhao^b†^, Lei Zhao^a†^，Qiyu Diao^C^，Lifeng Dong^C^*，Xiaolong Kang^a^**

1. **Supplementary Figure**

Supplementary Figure S1 (A). Scatterplot of PLS-DA scores and ranked validation plots. note: R^2^Y indicates the explanatory rate of the model, Q^2^Y is used to evaluate the predictive ability of the PLS-DA model, and when R^2^Y is greater than Q^2^Y, it means that the model is well established. (B). Differential metabolite ROC plot.
