# Supplementary material for: Effects of xylooligosaccharide supplementation on methane emissions, rumen microbiome, and serum metabolome in lactating Jersey cows
Source: Anim Biotechnol. 2026 Oct 22;38(1):2743468. doi: 10.1080/10495398.2026.2743468 (PMC13715329; doi:10.1080/10495398.2026.2743468)
Supplement: Supplementary file 2 [file LABT_A_2743468_SM3772.docx]

**Regulatory effects of Xylooligosaccharides on methane production, rumen microbiome and serum metabolism in lactating Jersey cow**

*Xiaonan Zhou^a†^, Lei Zhao^a†^，Qiyu Diao^C^，Lifeng Dong^C^*，Xiaolong Kang^a^* , Zhiyan Zhao^b†^*

**Supplementary Table S1-S5**

Supplementary Table S1 Ingredients fed to lactating Jersey cows during the study.

| Ingredient | g/kg DM |
| --- | --- |
| Corn silage | 423 |
| Alfalfa hay | 124 |
| Oat hay | 50 |
| Whole cottonseed | 26 |
| Beet pulp | 39 |
| Fat powder^1^ | 7 |
| Sodium bicarbonate | 3 |
| Molasses | 26 |
| Steam-flaked corn | 107 |
| Concentrate, commercial^2^ | 180 |
| Vitamin and mineral mix^3^ | 15 |

^1^Fat powder of palm oil Polyfat K200 (Britz (Xiamen) Trading Co., Ltd., Fujian province, China)

^2^Concentrate Dairy Jersey III (Guanghan Guoxiong Foodstuff Co., Ltd., Sichuan province, China): 27% corn, 32.4% soybean meal, 8% cottonseed meal, 15% rapeseed meal, 0.83% sodium bicarbonate, 5.5% corn gluten meal, 10.5% bypass soybean meal, 0.18% sodium chloride, 0.6% magnesium oxide

^3^Mineral mix contained 26.4% CP, 5.06% Ca, 10.7% Na, 6.8% K, 4.1% Mg, 0.26% S, 1.6% P, 417 mg/kg of Mn, 665 mg/kg of Zn, 229 mg/kg of Cu, 2,166 mg/kg of Fe, 24 mg/kg of Co, 14 mg/kg of I, 7.1 mg/kg of Se, 116,511 IU of vitamin A/kg, 13,100 IU of vitamin D3, and 1,164 IU of vitamin E/kg (DM basis).

| Supplementary Table S2 Barcode and primer information for the sample. | | | | |
| --- | --- | --- | --- | --- |
| Sample name | 515 Forward Primer Sequence | 806 Reverse Primer Sequence | Forward Primer barcode sequence | Reverse Primer barcode sequence |
| C.2562 | CCTAYGGGRBGCASCAG | GGACTACNNGGGTATCTAAT | TGACCA | ATTCCT |
| C.0651 | CCTAYGGGRBGCASCAG | GGACTACNNGGGTATCTAAT | ACAGTG | ATTCCT |
| C.0661 | CCTAYGGGRBGCASCAG | GGACTACNNGGGTATCTAAT | CTTGTA | GGTAGC |
| T.2138 | CCTAYGGGRBGCASCAG | GGACTACNNGGGTATCTAAT | ATCACG | ACTGAT |
| T.0686 | CCTAYGGGRBGCASCAG | GGACTACNNGGGTATCTAAT | ATGTCA | ATTCCT |
| T.0747 | CCTAYGGGRBGCASCAG | GGACTACNNGGGTATCTAAT | ACTTGA | ATTCCT |

| Supplementary Table S3 Data preprocessing statistics and quality control statistics. | | | | | | | | | | | |
| --- | --- | --- | --- | --- | --- | --- | --- | --- | --- | --- | --- |
| Sample name | Raw PE | Raw Tags | Clean Tags | Effective Tags | Base (nt) | AvgLen (nt) | Q20 | Q30 | GC (%) | Effective (%) |  |
| C.0661 | 80,234 | 79,572 | 78,892 | 62,760 | 26,391,101 | 421 | 98.22 | 94.23 | 52.92 | 78.22 |  |
| C.2562 | 82,800 | 82,576 | 81,962 | 64,171 | 26,966,618 | 420 | 98.15 | 94.06 | 52.31 | 77.5 |  |
| C.0651 | 91,209 | 90,690 | 89,971 | 69,386 | 29,037,889 | 418 | 98.2 | 94.21 | 52.7 | 76.07 |  |
| T.2138 | 78,634 | 78,584 | 78,232 | 61,712 | 25,851,178 | 419 | 98.27 | 94.39 | 53.76 | 78.48 |  |
| T.0686 | 76,499 | 76,423 | 76,003 | 56,619 | 23,480,387 | 415 | 98.28 | 94.4 | 55.18 | 74.01 |  |
| T.0747 | 88362 | 87447 | 86629 | 68236 | 28619230 | 419 | 98.17 | 94.07 | 53.12 | 77.22 |  |
| Note: Raw PE refers to the original PE reads from the sequencer; Raw Tags are the tag sequences obtained through assembly; Clean Tags are the sequences obtained after filtering out low-quality and short-length tags from the Raw Tags; Effective Tags are the tag sequences used for subsequent analysis after filtering out chimeras; Base refers to the number of bases in the final Effective Data; AvgLen denotes the average length of Effective Tags; Q20 and Q30 represent the percentage of bases with quality scores greater than 20 (sequencing error rate less than 1%) and 30 (sequencing error rate less than 0.1%) in Effective Tags; GC (%) indicates the GC content of Effective Tags; Effective (%) indicates the percentage of Effective Tags relative to the total number of Raw PE. | | | | | | | | | | | |

| Supplementary Table S4 Qualitative and quantitative list of 371 metabolites. | | | | | | | | | | | | | |
| --- | --- | --- | --- | --- | --- | --- | --- | --- | --- | --- | --- | --- | --- |
| ID | Name | Formula | Molecular Weight | RT [min] | neg_C_0661 | neg_C_2562 | neg_C_0651 | neg_T_2138 | neg_T_0686 | neg_T_0747 | neg_QC1 | neg_QC2 | neg_QC3 |
| Com_6_neg | Cholic acid | C24 H40 O5 | 408.28771 | 7.506 | 4524235494 | 3908501193 | 4998257110 | 6083290470 | 4282992378 | 5774532322 | 5537688448 | 4860358861 | 4525908448 |
| Com_30_neg | Uric acid | C5 H4 N4 O3 | 168.02817 | 1.781 | 1185736706 | 1251381076 | 1756246901 | 1309947872 | 1305508349 | 1336317531 | 1356516596 | 1397369092 | 1520010042 |
| Com_42_neg | D-(-)-Fructose | C6 H12 O6 | 180.06335 | 1.304 | 193875724.1 | 1136703715 | 165764296.3 | 240359996.4 | 1189012749 | 155555087.8 | 1052904913 | 1055867795 | 1009503670 |
| Com_68_neg | Salicylic acid | C7 H6 O3 | 138.03153 | 6.14 | 506961977.4 | 477174502.8 | 534662815.9 | 592340914.2 | 659363835 | 751985883.6 | 477169567.3 | 561274094.8 | 486421377.4 |
| Com_77_neg | 11,12-Epoxy-(5Z,8Z,11Z)-icosatrienoic acid | C20 H32 O3 | 320.23507 | 8.332 | 120762950.9 | 315888595.3 | 299194222.5 | 202845581.3 | 653268198.5 | 313768146 | 353696377.7 | 315829304.3 | 330971975.8 |
| Com_82_neg | Catechol | C6 H6 O2 | 110.03664 | 5.148 | 479621361.8 | 378320219.2 | 467515136.5 | 381935954.3 | 238992767.4 | 298275398.4 | 357817667.3 | 315667506.4 | 304796843.5 |
| Com_97_neg | (±)12(13)-DiHOME | C18 H34 O4 | 314.24572 | 8.702 | 326747309.2 | 274499271.1 | 358982727.5 | 385376903.8 | 398039934.7 | 489875527.4 | 371204953.5 | 345981871.7 | 329929405.7 |
| Com_110_neg | N-Isovalerylglycine | C7 H13 N O3 | 159.08947 | 5.298 | 196773684.6 | 208079676.1 | 227903763.1 | 285083764.9 | 198336767.8 | 433477278.1 | 235004214.9 | 231049778.2 | 252342922.4 |
| Com_111_neg | Uridine | C9 H12 N2 O6 | 244.06945 | 2.212 | 325735566.2 | 301228219.8 | 255833882.6 | 174632927.1 | 288116665.6 | 271235826 | 303721736 | 278049206.5 | 286408290.3 |
| Com_113_neg | Dodecanedioic acid | C12 H22 O4 | 230.15165 | 6.685 | 313833458 | 280385417.8 | 254023305.5 | 366324959.7 | 224810354.5 | 382757122.2 | 265279960 | 343630213.9 | 361593754.6 |
| Com_127_neg | 4-Oxoproline | C5 H7 N O3 | 129.04255 | 1.871 | 197636837 | 81158567.65 | 278384300 | 231893122.9 | 226381029.5 | 95531434.56 | 164338188.1 | 186941987.9 | 165872680.2 |
| Com_138_neg | L-Phenylalanine | C9 H11 N O2 | 165.07894 | 4.885 | 187822123.7 | 235728583.3 | 176486380.9 | 279156350.7 | 263645273.5 | 264238431.4 | 214412154.7 | 223799021.3 | 233965681.7 |
| Com_203_neg | 3-Hydroxybutyric acid | C4 H8 O3 | 104.04743 | 2.782 | 109113421.1 | 157629668.5 | 136166432.1 | 165314878.9 | 95326487.24 | 109580370.7 | 116934901.6 | 112324551 | 109199655.3 |
| Com_208_neg | 4-Methylcatechol | C7 H8 O2 | 124.05238 | 5.865 | 151479569.7 | 70336697.12 | 93910021.79 | 85133657.72 | 35144524.24 | 72616134.71 | 81332860.19 | 84501750.22 | 82760625.9 |
| Com_219_neg | 3-Indoxyl sulphate | C8 H7 N O4 S | 213.00957 | 5.373 | 143480957.9 | 103206340.5 | 109714410.3 | 136112249.1 | 80807005.04 | 111488257.9 | 123092706 | 114393285.3 | 120255254.5 |
| Com_267_neg | Citric acid | C6 H8 O7 | 192.02696 | 2.193 | 113803581.2 | 72595865.07 | 74795029.34 | 18791554.97 | 71591786.9 | 34657886.86 | 63078750.76 | 68237754.2 | 70711953.16 |
| Com_332_neg | Adrenic acid | C22 H36 O2 | 332.27151 | 10.582 | 55652051.13 | 82427581.63 | 47718415.69 | 75776534.61 | 69060828.66 | 54088336.7 | 89180681.72 | 73798505.3 | 75677311.25 |
| Com_333_neg | 2'-Deoxyuridine | C9 H12 N2 O5 | 228.07466 | 2.899 | 85813240.25 | 86766876.81 | 67646450.41 | 70550152.19 | 75519269.52 | 63258822.79 | 76213275.27 | 61022247.46 | 66064946.95 |
| Com_336_neg | 3-Hydroxy-L-proline | C5 H9 N O3 | 131.0582 | 2.371 | 78176571.13 | 62122821.2 | 47652147.68 | 66078266.04 | 31152174.97 | 110585910.9 | 68398725.2 | 63676888.78 | 37334475.59 |
| Com_341_neg | 2,4-Dihydroxybenzoic acid | C7 H6 O4 | 154.02658 | 5.901 | 73892873.05 | 52676003 | 87436410.77 | 69833009.4 | 23612845.36 | 52637354.67 | 62875602.42 | 53272135.07 | 56881455.2 |
| Com_354_neg | 4-Nitrophenol | C6 H5 N O3 | 139.02692 | 5.888 | 62259690.3 | 58986041.21 | 64182259.11 | 72196726.79 | 84836457.17 | 103303994.2 | 57729168.37 | 59876681.55 | 60271928.53 |
| Com_415_neg | Thymidine | C10 H14 N2 O5 | 242.0903 | 4.843 | 66504478.22 | 61065158.98 | 48410154.76 | 45407904.32 | 67680234.3 | 58110028.6 | 56155056.31 | 54532099.39 | 50002451.73 |
| Com_439_neg | N-Tigloylglycine | C7 H11 N O3 | 157.07381 | 5.238 | 48643388.95 | 37315182.5 | 64275530.15 | 68349524.34 | 33526220.77 | 75081051.71 | 53312474.48 | 53040556.87 | 53623907.02 |
| Com_446_neg | 5-Aminovaleric acid | C5 H11 N O2 | 117.07891 | 1.717 | 36804573.33 | 61574225.18 | 26048624.97 | 45895721.29 | 53736921.12 | 33215541 | 61283329.37 | 38530255.02 | 54605472.26 |
| Com_532_neg | N-Acetylornithine | C7 H14 N2 O3 | 174.10027 | 1.434 | 25002639.73 | 31312417.48 | 16717370.81 | 37262931.42 | 39052283.97 | 64096489.82 | 39662515.86 | 34274522.52 | 40206768.91 |
| Com_587_neg | L-Histidine | C6 H9 N3 O2 | 155.0695 | 1.29 | 26058822.87 | 44164671.73 | 13504235.58 | 19506484.72 | 31220750.43 | 30241307.38 | 25603265.62 | 23687306.33 | 23260414.66 |
| Com_600_neg | D-(-)-Glutamine | C5 H10 N2 O3 | 146.06902 | 1.338 | 20871812.39 | 42951055.1 | 26652628.63 | 16472686.08 | 31165735.07 | 29742661.34 | 30173711.91 | 28374407.11 | 43958959.67 |
| Com_848_neg | L-Threonic acid | C4 H8 O5 | 136.03711 | 1.362 | 21023424.03 | 23219656.95 | 16107954.08 | 17429821.17 | 34301102.88 | 22921648.34 | 25964878.77 | 26185253.91 | 25096341.63 |
| Com_1076_neg | 3-Methyladipic acid | C7 H12 O4 | 160.07343 | 5.475 | 20870046.63 | 20115480.42 | 14056288.54 | 14069495.25 | 16753362.71 | 26333237.97 | 17202798.59 | 19872578.32 | 19963562.68 |
| Com_1256_neg | β-D-Glucopyranuronic acid | C6 H10 O7 | 194.04245 | 5.429 | 17091892.53 | 16870034.5 | 11678104.98 | 10615629.25 | 11983743.15 | 9918946.063 | 12957181.06 | 12487868.97 | 14639367.01 |
| Com_1263_neg | 2-Furoic acid | C5 H4 O3 | 112.01609 | 1.77 | 16986672.02 | 10887028.15 | 10287847.52 | 2563243.744 | 9013726.103 | 4774705.655 | 9352388.606 | 9107482.348 | 8230407.649 |
| Com_1283_neg | 1,2,3-cyclopropanetricarboxylic acid | C6 H6 O6 | 174.01625 | 2.265 | 15827114.72 | 14195303.59 | 17318111.17 | 4733691.968 | 8299164.916 | 6168539.498 | 13305494.96 | 11385720.4 | 10368984.13 |
| Com_1647_neg | Ethylmalonic acid | C5 H8 O4 | 132.04221 | 4.994 | 12658720.94 | 9986682.964 | 9771110.941 | 9947264.707 | 9517629.585 | 13470321.47 | 10917650.73 | 9926637.022 | 11358754.92 |
| Com_1683_neg | N-Acetyl-DL-norvaline | C7 H13 N O3 | 159.08949 | 4.961 | 7434418.174 | 8121041.06 | 6906527.85 | 12643768.39 | 13238129.63 | 14429370.06 | 12745819.14 | 9592040.267 | 10353471.28 |
| Com_1812_neg | 2-Isopropylmalic acid | C7 H12 O5 | 176.06837 | 4.855 | 10661336.67 | 10727115.38 | 9317803.054 | 10427516.34 | 10749311.68 | 12838732.24 | 11587306.76 | 11189876.39 | 9107256.866 |
| Com_2110_neg | N-Acetylglycine | C4 H7 N O3 | 117.04255 | 1.563 | 6230944.852 | 4909395.946 | 6985971.8 | 9195757.303 | 5208709.036 | 11712709.09 | 6464155.761 | 7028389.689 | 6889502.003 |
| Com_2739_neg | 4-Pyridoxic acid | C8 H9 N O4 | 183.05314 | 3.56 | 5875772.856 | 5514468.898 | 5074256.774 | 7176359.555 | 6033056.515 | 6406146.735 | 5367732.363 | 5858998.016 | 5533073.562 |
| Com_3165_neg | Taurine | C2 H7 N O3 S | 125.01474 | 1.256 | 3548574.711 | 2952579.871 | 5040996.32 | 4371700.067 | 5721300.531 | 4863522.183 | 2636519.677 | 5102348.967 | 5325671.894 |
| Com_3442_neg | Acetaminophen glucuronide | C14 H17 N O8 | 327.0956 | 3.862 | 4239300.326 | 2458050.065 | 2811463.978 | 3026668.359 | 1862408.93 | 3023741.377 | 1993047.237 | 2907567.521 | 3107305.623 |
| Com_4083_neg | Xanthosine | C10 H12 N4 O6 | 284.07561 | 4.866 | 962680.0353 | 2442931.454 | 2339565.753 | 3653337.537 | 2402398.107 | 2745840.297 | 2292278.233 | 2156928.365 | 2231945.196 |
| Com_4402_neg | N2-Methylguanosine | C11 H15 N5 O5 | 297.10726 | 4.777 | 2716263.982 | 1729633.289 | 2239047.576 | 2283217.093 | 2334148.927 | 2441432.095 | 2136235.624 | 2082261.65 | 2360185.517 |
| Com_18_neg | Phenylacetylglycine | C10 H11 N O3 | 193.07378 | 5.523 | 2688988614 | 2214865909 | 2049852243 | 2455261111 | 2555601444 | 2540072316 | 2165559769 | 2413388231 | 2396261232 |
| Com_502_neg | Glycyl-L-leucine | C8 H16 N2 O3 | 188.11613 | 5.024 | 4715525.258 | 8494886.44 | 55179634.98 | 8278315.029 | 9739914.783 | 5734982.193 | 14659362.39 | 16631119.17 | 16069917.56 |
| Com_537_neg | Guanosine | C10 H13 N5 O5 | 283.09168 | 3.294 | 6696710.251 | 50382096.79 | 337474.8024 | 4282846.927 | 9293121.688 | 14326634.79 | 13469340.58 | 11780479.73 | 12437912.33 |
| Com_387_neg | Glycerophospho-N-palmitoyl ethanolamine | C21 H44 N O7 P | 453.28574 | 9.397 | 42252794.14 | 59348071.71 | 51345623.66 | 61242545.76 | 49343443.65 | 51670757.92 | 74052824.21 | 62704959.59 | 60482230.75 |
| Com_1059_neg | Thromboxane B2 | C20 H34 O6 | 370.23534 | 6.523 | 7868155.345 | 12709643.47 | 17929990.67 | 12067728.57 | 26094207.13 | 15887823.21 | 16449321.28 | 14430755.3 | 16351862.67 |
| Com_501_neg | 2-Hydroxyhippuric acid | C9 H9 N O4 | 195.05301 | 5.102 | 53502352.44 | 24306513.28 | 31650114.92 | 42005875 | 29999585.17 | 48514148.95 | 34483157.7 | 41174093.08 | 37088541.56 |
| Com_1418_neg | Adenosine 3'5'-cyclic monophosphate | C10 H12 N5 O6 P | 329.05265 | 3.7 | 4899232.78 | 7905675.43 | 4057266.563 | 17736185.23 | 2212476.064 | 9859270.292 | 5808644.427 | 7337566.81 | 5661699.845 |
| Com_1474_neg | Carnosine | C9 H14 N4 O3 | 226.10649 | 1.316 | 14531373.58 | 10408585.65 | 11526086.43 | 8168060.984 | 15526629.9 | 12054759.15 | 14274680.74 | 13999163.34 | 15016024.27 |
| Com_3515_neg | N4-Acetylcytidine | C11 H15 N3 O6 | 285.09602 | 4.874 | 2898437.807 | 3183613.641 | 2034248.108 | 4252585.424 | 4011024.9 | 5253641.266 | 3450807.382 | 3448274.321 | 3281242.288 |
| Com_1127_neg | Docosahexaenoic Acid | C22 H32 O2 | 328.24028 | 9.867 | 16834910.22 | 18053761.05 | 8167825.336 | 18158877.14 | 21407591.7 | 14879737.8 | 20469658.49 | 17553522.91 | 17763711.86 |
| Com_3130_neg | Levothyroxine | C15 H11 I4 N O4 | 776.68766 | 6.379 | 3033926.523 | 5110677.958 | 2351538.29 | 2948886.442 | 3053851.783 | 3502030.746 | 4197772.893 | 3484049.879 | 3063059.529 |
| Com_10_neg | Hippuric acid | C9 H9 N O3 | 133.05257 | 5.384 | 4540563503 | 3573321628 | 3407218358 | 4069610821 | 3379147264 | 4679549329 | 3703435594 | 4146457849 | 3992692931 |
| Com_12_neg | LPC 16:0 | C24 H50 N O7 P | 541.33829 | 9.438 | 3205636993 | 3943189945 | 3402840728 | 3923325829 | 3926503807 | 3782945867 | 4185086685 | 3840013919 | 3801285618 |
| Com_14_neg | LPC 18:0 | C26 H54 N O7 P | 523.36432 | 10.226 | 2559038314 | 2371622050 | 2067874307 | 2661267075 | 2808393276 | 2050807530 | 3596981716 | 2148161282 | 2297194738 |
| Com_20_neg | LPC 18:1 | C26 H52 N O7 P | 567.35402 | 9.646 | 1992677637 | 2170857300 | 2256507761 | 1838855378 | 2086663726 | 2039180125 | 2484853431 | 2079359050 | 2020516888 |
| Com_24_neg | Stearic acid | C18 H36 O2 | 284.27147 | 11.201 | 1360057638 | 2221125782 | 1062370151 | 1686369217 | 1703363320 | 1368071681 | 1747621201 | 1695224802 | 2209411854 |
| Com_28_neg | 4-Methylphenol | C7 H8 O | 108.05754 | 6.075 | 1367841121 | 1432989495 | 1905453271 | 531214357 | 1489579457 | 1220977731 | 1506470212 | 1300397767 | 1284016242 |
| Com_35_neg | 2-Ketohexanoic acid | C6 H10 O3 | 130.06296 | 5.736 | 1337330016 | 1505834692 | 987638186.4 | 1506947903 | 1433065917 | 1559858266 | 1334829760 | 1400020707 | 1515153429 |
| Com_37_neg | Elaidic acid | C18 H34 O2 | 282.25578 | 10.572 | 864140845.1 | 1107584637 | 825410215.6 | 1431193559 | 1060319480 | 919540454.5 | 1453159375 | 1144557945 | 1290577944 |
| Com_44_neg | PC (16:0/18:2) | C42 H80 N O8 P | 803.56858 | 11.317 | 960745954.1 | 934289042.4 | 874134476.8 | 921835600.7 | 374151862.8 | 1103899146 | 1080094520 | 886374980.7 | 537140079 |
| Com_49_neg | LPC 18:2 | C26 H50 N O7 P | 565.33834 | 8.924 | 856122078.7 | 859717393.2 | 826416750 | 499356413.2 | 1071193869 | 862343830.4 | 984162532.4 | 714662037.4 | 891694116.3 |
| Com_55_neg | SM (d14:1/20:0) | C39 H79 N2 O6 P | 748.57418 | 10.218 | 608637134.7 | 451431650.1 | 752495778.2 | 524913127.7 | 570311601.4 | 902249371.2 | 799950098.1 | 624091369.8 | 484590014.1 |
| Com_56_neg | LPC 20:3 | C28 H52 N O7 P | 591.35412 | 9.39 | 585356915.4 | 781904547.7 | 731448057.3 | 312051480.3 | 651796073.5 | 495491578.6 | 710328014.6 | 582788210 | 537705444.8 |
| Com_58_neg | Deoxycholic acid | C24 H40 O4 | 392.29284 | 8.263 | 718412676.9 | 398978768.1 | 679332458.5 | 591959319 | 347346667.6 | 620540721.6 | 670431913.3 | 589435413.8 | 617985469.7 |
| Com_73_neg | LPC 17:0 | C25 H52 N O7 P | 555.35397 | 9.838 | 446100679.6 | 442957403.1 | 478074500.4 | 440159612.6 | 472975096.1 | 487391823.4 | 577919996.9 | 473735784.4 | 435939529.1 |
| Com_74_neg | Dihydroroseoside | C19 H32 O8 | 388.20994 | 5.819 | 551454879.8 | 186654838.1 | 386807727.4 | 562156922.4 | 360359994.8 | 391111186 | 390701220.5 | 389209300.7 | 435664139.5 |
| Com_75_neg | DL-Tryptophan | C11 H12 N2 O2 | 158.08436 | 5.138 | 303867837.2 | 443422520.5 | 304099686.8 | 313827364.3 | 660989016.1 | 474535611.3 | 352954684.8 | 381736902.2 | 360858092.1 |
| Com_76_neg | LPC 20:4 | C28 H50 N O7 P | 589.33852 | 9.039 | 382125973.1 | 553705295.4 | 408080111.9 | 335188640.7 | 525773709.1 | 351595783.9 | 392243331.6 | 416371606.1 | 381342732.4 |
| Com_89_neg | Azelaic acid | C9 H16 O4 | 188.10466 | 5.925 | 408144242.9 | 348489554.8 | 325352479.1 | 449296027.9 | 349876549.6 | 560705695.6 | 402705635.5 | 429137987.5 | 416264766.7 |
| Com_92_neg | Taurochenodeoxycholic acid | C26 H45 N O6 S | 499.29699 | 11.211 | 273859357.8 | 439424549.4 | 337195426.6 | 239274504.1 | 489166673 | 60339852.63 | 428435344.2 | 258247955.7 | 301499602.5 |
| Com_99_neg | 8-iso-15-keto Prostaglandin F2α | C20 H32 O5 | 334.21447 | 7.632 | 215290633.3 | 179543687.5 | 207968018.2 | 229470109.8 | 244690263.7 | 487205375.5 | 253550753.2 | 228172842.4 | 250386819 |
| Com_100_neg | PC (18:1/18:2) | C44 H82 N O8 P | 829.58473 | 11.899 | 333490902 | 377086831.9 | 349902756.7 | 389739192.5 | 331385807.1 | 357907831.1 | 386673148.9 | 334812175 | 300868358.2 |
| Com_102_neg | LPC 16:1 | C24 H48 N O7 P | 539.32272 | 8.854 | 297993867.3 | 354695114 | 344113601.5 | 371411399.2 | 325317927.7 | 317560407 | 377329789.9 | 313638418.5 | 333301730.5 |
| Com_109_neg | 2-Hydroxy-2-methylbutanoic acid | C5 H10 O3 | 118.063 | 5.11 | 336313399.6 | 248166125.9 | 250583085.4 | 285574313.2 | 146037316.9 | 167689455.9 | 225230166.9 | 269561405.3 | 220216350.2 |
| Com_123_neg | Sulfaquinoxaline | C14 H12 N4 O2 S | 300.07053 | 4.828 | 279457886.8 | 117987338 | 262243266.7 | 306733208.7 | 344641452.2 | 107301766.4 | 237978647.8 | 234434309 | 231003896.9 |
| Com_126_neg | 5,6-dihydroxyindole | C8 H7 N O2 | 149.04749 | 5.577 | 272190259 | 149582123.2 | 175102538.5 | 203079617.1 | 77739827.44 | 167527474 | 161037638.7 | 168023757.4 | 175361392.6 |
| Com_133_neg | LysoPE 18:0 | C23 H48 N O7 P | 481.317 | 9.439 | 207852520.9 | 218564482.7 | 212865584.1 | 237775492.2 | 257853778.8 | 243800071.9 | 258166874.5 | 221861904.4 | 235916774.6 |
| Com_134_neg | 8Z,11Z,14Z-Eicosatrienoic acid | C20 H34 O2 | 306.2558 | 10.312 | 189307869.1 | 253142259.6 | 181803764.4 | 166599164.8 | 239912966.6 | 150611165.5 | 234104213.7 | 197933625.9 | 215821658 |
| Com_147_neg | (±)9-HpODE | C18 H32 O4 | 312.23012 | 8.323 | 199834439.9 | 191369376.6 | 217327490.7 | 272000111.9 | 205238591 | 285965013.5 | 199849186 | 176022316.3 | 217517397.9 |
| Com_157_neg | methadone-d9 | C21 H18 [2]H9 N O | 317.9549 | 1.151 | 181930091.4 | 171236607.7 | 226646010.3 | 208465899.9 | 69974823.41 | 270984669.4 | 226646305.1 | 187607524.3 | 242788173.2 |
| Com_163_neg | PE (16:0/18:2) | C39 H74 N O8 P | 715.51634 | 11.053 | 118975120.3 | 98652032.31 | 146095258.2 | 75904028.37 | 108961902.5 | 267139444.9 | 117139433.2 | 137658026.8 | 71715017.06 |
| Com_166_neg | LPC 14:0 | C22 H46 N O7 P | 513.30683 | 8.616 | 151038552.5 | 181544858.4 | 183710067.7 | 173536632.6 | 146786114.5 | 227523584.5 | 209116867.1 | 198431555.5 | 176532599.7 |
| Com_176_neg | Arachidic acid | C20 H40 O2 | 312.3028 | 11.358 | 188589524.6 | 154809715.4 | 137189630.2 | 195108334.1 | 107634834 | 152064688.5 | 188818895.2 | 162371283.2 | 157536445.8 |
| Com_186_neg | 3-(methylsulfanyl)-5H-[1,2,4]triazino[5,6-b]indole | C10 H8 N4 S | 216.04558 | 5.566 | 95457661.17 | 116618993.3 | 114124570 | 110398086.3 | 69694795.9 | 227349471.7 | 127443315.2 | 101270809.6 | 134381598.7 |
| Com_196_neg | PC (16:0/20:4) | C44 H80 N O8 P | 827.56882 | 11.07 | 127199197.6 | 121932300.2 | 109250137.4 | 98603517.85 | 76825474.76 | 206437169.4 | 105131589 | 154948021.8 | 106821655.6 |
| Com_197_neg | 13(S)-HOTrE | C18 H30 O3 | 294.21952 | 7.819 | 121732848.9 | 111575830.6 | 129069416 | 183304699.2 | 111659325.7 | 167693440.9 | 142785297.2 | 129848831.1 | 140260557 |
| Com_198_neg | Lysopc 17:0 | C25 H52 N O7 P | 509.34829 | 10.225 | 111128895.3 | 135901262.3 | 127557816.2 | 126982395.7 | 131900368.4 | 124100475.4 | 162272062.1 | 137127548.3 | 129826417 |
| Com_214_neg | LPC 22:4 | C30 H54 N O7 P | 617.36978 | 9.706 | 98479096.24 | 150753200.4 | 94495726.98 | 119066639.7 | 138301812.6 | 103634837.2 | 131614706.6 | 111606586.9 | 105980825.6 |
| Com_233_neg | PC (14:1e/18:2) | C40 H76 N O7 P | 759.54241 | 9.547 | 131658000.8 | 65801536.21 | 104941397.4 | 97006476.32 | 69041351.24 | 149872216.5 | 101375509.7 | 144322408.1 | 112341335.5 |
| Com_235_neg | 2-{2-[2,5-di(methoxycarbonyl)anilino]-2-oxoethoxy}acetic acid | C14 H15 N O8 | 325.07967 | 5.141 | 111174458.8 | 136020861.2 | 84871859.05 | 100884202.6 | 107076248.4 | 138670336.5 | 114648811.2 | 122149281.4 | 115630418.3 |
| Com_253_neg | FAHFA (18:0/20:2) | C38 H70 O4 | 590.5256 | 11.208 | 77235427.36 | 125381086.7 | 36563901.83 | 52519142.36 | 96983506.31 | 51487953.51 | 128563608.1 | 74593640.34 | 105566271 |
| Com_268_neg | 16-Hydroxyhexadecanoic acid | C16 H32 O3 | 254.22453 | 9.788 | 77561078.08 | 89575345.92 | 76147588.69 | 131474965.5 | 97113534.73 | 91178112.45 | 116307330.4 | 106751250.8 | 112634502.1 |
| Com_277_neg | LPC 20:5 | C28 H48 N O7 P | 587.32292 | 8.611 | 60345060.86 | 110361297.6 | 73217191.44 | 57023809.12 | 71816344.85 | 66654913.45 | 85206032.76 | 76162956.59 | 66205808.46 |
| Com_285_neg | SM (d14:1/18:0) | C37 H75 N2 O6 P | 720.54289 | 11.062 | 84091315.81 | 104640487.3 | 92511240.72 | 69306334.59 | 95412984.33 | 82368492.67 | 102392682.2 | 73551422.15 | 87408533.53 |
| Com_295_neg | 20-Hydroxy-(5Z,8Z,11Z,14Z)-eicosatetraenoic acid | C20 H32 O3 | 320.23506 | 9.4 | 77754698.22 | 91647733.18 | 80591083.17 | 14379691.23 | 99389013.28 | 89940951.56 | 103341069 | 98106097.87 | 94854457.76 |
| Com_297_neg | Docosapentaenoic acid | C22 H34 O2 | 330.25585 | 10.138 | 70792766.83 | 86734270.5 | 60058119.31 | 105838338 | 84487207.51 | 64599328.86 | 101926085 | 75599154.82 | 90882900.99 |
| Com_299_neg | Aldosterone | C21 H28 O5 | 360.19712 | 11.955 | 91181714.42 | 76423858.14 | 95111337.06 | 81085123.74 | 83868287.79 | 112143541.7 | 100845140.2 | 64127459.67 | 70401883.92 |
| Com_303_neg | PC (16:0/20:5) | C44 H78 N O8 P | 825.55332 | 8.809 | 71430639.9 | 70251885.45 | 59000718.28 | 64569833.52 | 71222486.83 | 64869416.98 | 99075682.66 | 78109357.04 | 56843771.51 |
| Com_305_neg | LPC 20:2 | C28 H54 N O7 P | 593.36991 | 9.881 | 80284547.94 | 96840709.47 | 88161896.81 | 45378249.56 | 85452607.08 | 53098242.33 | 93749863.23 | 73372333.96 | 67023981.67 |
| Com_340_neg | cis-5,8,11,14,17-Eicosapentaenoic acid | C20 H30 O2 | 302.22449 | 9.494 | 62808921.25 | 79143483.32 | 69131411.57 | 72648364.75 | 84475190.79 | 75954843.07 | 88097063.92 | 82386845.46 | 80072437.03 |
| Com_343_neg | LPC 22:5 | C30 H52 N O7 P | 615.35429 | 9.065 | 84241558.37 | 6132715.136 | 5121624.188 | 6164934.403 | 5591527.963 | 6254841.099 | 3642683.654 | 5242180.441 | 4540015.253 |
| Com_353_neg | Chenodeoxycholic Acid | C24 H40 O4 | 438.29829 | 8.203 | 61433084.61 | 42497256.57 | 12406784.01 | 16061334.54 | 36787035.17 | 82452094.49 | 82614956.11 | 61913886.63 | 71215397.8 |
| Com_358_neg | 2-Methylbutyl beta-D-glucopyranoside | C11 H22 O6 | 296.14711 | 5.041 | 78996487.4 | 50738007.23 | 65057766.15 | 60660103.84 | 50146862.99 | 89071383.71 | 64833665.51 | 69056113.51 | 65712801.9 |
| Com_366_neg | SM (d14:2/20:0) | C39 H77 N2 O6 P | 746.5588 | 11.318 | 71588451.11 | 68705874.96 | 79227223.49 | 64348068.29 | 69594558.51 | 73992906.16 | 69271106.24 | 58423839.76 | 51740278.41 |
| Com_369_neg | PC (14:1e/20:4) | C42 H76 N O7 P | 783.54292 | 8.225 | 59250555.35 | 50239563.98 | 42773456.81 | 37734553.26 | 50702175.96 | 64139362.26 | 76816818.2 | 81359701.93 | 47998854.01 |
| Com_372_neg | Allantoin | C4 H6 N4 O3 | 158.04384 | 1.368 | 75178542.02 | 59718395.91 | 56926868.38 | 63318761.39 | 46137966.81 | 67439328.63 | 62376834.41 | 65122331.07 | 65479296.8 |
| Com_383_neg | PC (16:0/18:1) | C42 H82 N O8 P | 805.57533 | 11.295 | 32554566.93 | 53225649.92 | 65968299.46 | 84226398.4 | 57112930.74 | 81881348.05 | 61524183.47 | 57810676.94 | 49947635.68 |
| Com_395_neg | PE (18:1/18:2) | C41 H76 N O8 P | 741.53215 | 11.506 | 51492991.23 | 38512385.89 | 71919976.42 | 52327382.06 | 41743797.5 | 87751963.8 | 48711910.02 | 52210592.82 | 31618338.01 |
| Com_401_neg | LPC 12:0 | C20 H42 N O7 P | 485.27556 | 7.808 | 41253510.46 | 30280277.88 | 38974464.99 | 31141888.48 | 36947694.27 | 88551818.81 | 50900448.7 | 42264534.02 | 47960341.8 |
| Com_409_neg | Levodopa | C9 H11 N O4 | 197.06864 | 6.148 | 46272898.7 | 57437600.37 | 42857317.7 | 60481007.37 | 60064030.98 | 87393704.19 | 52602656.71 | 58488282.88 | 58091559.77 |
| Com_410_neg | DL-4-Hydroxyphenyllactic acid | C9 H10 O4 | 136.05248 | 5.19 | 53942965.08 | 68744069.93 | 44629844.79 | 47444264.55 | 63513100.96 | 62701599.37 | 54212713.72 | 53504871.84 | 53978733.54 |
| Com_413_neg | PE (16:1e/18:2) | C39 H74 N O7 P | 699.5214 | 11.638 | 42956325.7 | 34725491.13 | 40269095.25 | 49909476.2 | 37528461 | 86813186.17 | 38429934.61 | 45266433.42 | 36675867.1 |
| Com_419_neg | 3-(4-Hydroxyphenyl)propionic acid | C9 H10 O3 | 120.05741 | 5.644 | 66042429.7 | 45552878.2 | 58138114.53 | 62584522.06 | 36748942.22 | 60983731.49 | 51821855.12 | 51081025.55 | 55164546.77 |
| Com_422_neg | 7-Hydroxy-3,4-dihydrocarbostyril | C9 H9 N O2 | 163.06319 | 5.698 | 53636105.82 | 64035677.31 | 37631448.39 | 76219509.91 | 43440966.9 | 71525219.3 | 55275248.76 | 50590159.49 | 59852245.13 |
| Com_431_neg | Indole-2-carboxylic acid | C9 H7 N O2 | 161.04745 | 5.831 | 39914945.49 | 65541402.69 | 42277997.31 | 42905524.14 | 38758772.84 | 70395068.22 | 47695035.03 | 54085125.22 | 54416111.18 |
| Com_447_neg | SM (d14:1/19:0) | C38 H77 N2 O6 P | 734.55864 | 11.744 | 55898877.88 | 60782209.6 | 62069166.58 | 31348960.45 | 63974048.71 | 54461524.28 | 59296048.8 | 50848071.27 | 40818648.66 |
| Com_460_neg | PE (16:1e/20:4) | C41 H74 N O7 P | 723.52064 | 11.227 | 37461904.22 | 55575963.32 | 43178788.01 | 36417955.3 | 70269196.17 | 66445835.19 | 42854417.15 | 41593558.94 | 38560540.42 |
| Com_473_neg | Cer-NS (d18:1/16:0) | C34 H67 N O3 | 537.51223 | 10.26 | 42003713.31 | 38850795.73 | 55611453.96 | 50402093.77 | 52059141.32 | 73199258.78 | 39979122.83 | 50056387.53 | 59295637.84 |
| Com_475_neg | PE (14:1e/18:3) | C37 H68 N O7 P | 669.47303 | 11.239 | 12161175.1 | 41200818.48 | 21119093.04 | 36297914.13 | 67177701.82 | 14674838.1 | 32755454.13 | 42471855.37 | 64253931.7 |
| Com_492_neg | Octanedioic acid | C8 H14 O4 | 174.08909 | 5.698 | 54317089.08 | 50824427.59 | 54146987.79 | 56332678.94 | 46913813.25 | 68120364.86 | 47663702.46 | 49287260.62 | 55631458 |
| Com_498_neg | Valproic acid | C8 H16 O2 | 144.11491 | 5.926 | 52055738.55 | 44326290.17 | 42907807.79 | 60184488.23 | 44260040.67 | 65710107.32 | 55737349.66 | 56176583.31 | 52822082.02 |
| Com_504_neg | 11(E)-Eicosenoic Acid | C20 H38 O2 | 310.28726 | 11.341 | 28734802.3 | 36690470.01 | 28990343.29 | 62152642.6 | 24303548.22 | 30073463.29 | 43727391.57 | 36601147.08 | 37611880.27 |
| Com_517_neg | 3-Methyl-2-oxobutanoic acid | C5 H8 O3 | 116.04737 | 4.707 | 51554093.79 | 52825263.61 | 40782284.18 | 55879717.97 | 55205506.91 | 60344540.51 | 46258678.41 | 55499999 | 54487473.86 |
| Com_520_neg | Heptanoic acid | C7 H14 O2 | 130.09928 | 6.198 | 37369351.45 | 40226369.97 | 24658906.38 | 30648811.77 | 44387132.76 | 66469019.72 | 36041042.45 | 33114829.74 | 40787373.33 |
| Com_529_neg | 2-Furoylglycine | C7 H7 N O4 | 169.0374 | 4.894 | 50152380.57 | 26106208.59 | 38808629.51 | 52793791.14 | 30274279.14 | 37726277.56 | 37272785.28 | 37239310.27 | 36397118.03 |
| Com_536_neg | PE (16:2e/18:2) | C39 H72 N O7 P | 697.50567 | 10.486 | 22130463.05 | 21189999.62 | 21226384.76 | 31302377.38 | 15026109.48 | 63833985.35 | 12990755.43 | 19551435.28 | 21146656.94 |
| Com_541_neg | FAHFA (6:0/18:0) | C24 H46 O4 | 398.3396 | 11.026 | 33987684.62 | 49771513.39 | 22266048.73 | 29069254.85 | 31555300.26 | 41758582.14 | 35760321.37 | 41280469.55 | 40099906.92 |
| Com_547_neg | (±)9(10)-DiHOME | C18 H34 O4 | 314.24572 | 7.564 | 29145392.36 | 39075442.69 | 43906876.15 | 56139543.78 | 28000550.21 | 57568460 | 41471560.79 | 40454659.69 | 33087491.18 |
| Com_560_neg | 11(Z),14(Z)-Eicosadienoic Acid | C20 H36 O2 | 308.27154 | 10.799 | 36147711.46 | 40999707.04 | 31808406.35 | 46587363.72 | 41501106.47 | 29739329.99 | 48666531.47 | 41064520.33 | 44689206.71 |
| Com_592_neg | Lauric acid ethyl ester | C14 H28 O2 | 228.20896 | 9.562 | 26554653.97 | 43779676.5 | 26393970.18 | 43507811.84 | 46608001.76 | 35995693.4 | 43475103.31 | 43198232.62 | 43542196.66 |
| Com_597_neg | Citrulline | C6 H13 N3 O3 | 175.09563 | 1.356 | 24854393.53 | 24332670.91 | 21483299.05 | 19052281.06 | 51433125.13 | 31170720.46 | 30530520.17 | 31844974.98 | 32143941.63 |
| Com_603_neg | Prostaglandin H2 | C20 H32 O5 | 352.22502 | 7.194 | 30212159.85 | 35778462.86 | 28970832.69 | 26062088.48 | 48052136.41 | 54088764.96 | 34764961.12 | 36573880.54 | 34432682.14 |
| Com_612_neg | Prostaglandin D2 | C20 H32 O5 | 334.21443 | 7.194 | 29984714.4 | 35292795.55 | 28071398.39 | 25834245.65 | 47759000.84 | 53240018.51 | 34356477.2 | 36074179.64 | 33738272.71 |
| Com_614_neg | Phenylpyruvic acid | C9 H8 O3 | 164.04729 | 5.653 | 31226721.43 | 36533165.52 | 29937301.87 | 23315975.47 | 34698570.33 | 53169150.94 | 38330524.66 | 29805386.61 | 33582252.78 |
| Com_632_neg | SM (d14:2/22:0) | C41 H81 N2 O6 P | 774.59036 | 10.771 | 29473965.66 | 26041094.61 | 26032552.69 | 27687356.99 | 24328084.1 | 45626124.15 | 41155859.49 | 28152667.82 | 24549477.33 |
| Com_647_neg | 5-Sulfosalicylic acid | C7 H6 O6 S | 217.98799 | 5.069 | 38691805.48 | 31554501.01 | 34655074 | 35714751.81 | 38753036.25 | 36078560.24 | 31556812.13 | 34311624.68 | 43767528.16 |
| Com_651_neg | Ascorbic acid | C6 H8 O6 | 176.03195 | 1.434 | 4695861.272 | 39442345.76 | 3523593.434 | 3991186.572 | 4479905.073 | 31585860.26 | 4990493.613 | 3604940.146 | 3746307.824 |
| Com_661_neg | 2-Hydroxyisocaproic Acid | C6 H12 O3 | 132.07852 | 5.72 | 36733655.8 | 38316487.34 | 36835130.23 | 40801204.27 | 6035339.677 | 28236249.36 | 31952336.93 | 32895533.67 | 40173797.12 |
| Com_662_neg | Hexadecanedioic acid | C16 H30 O4 | 286.21425 | 7.763 | 37444641.9 | 34169723.49 | 36119880.23 | 36735394.62 | 28472597.23 | 29240854.38 | 33440206.99 | 32144180.75 | 29115719.5 |
| Com_664_neg | DL-β-Leucine | C6 H13 N O2 | 131.09453 | 2.742 | 32302478.29 | 36408099.61 | 26984482.71 | 33914564.78 | 45455804.03 | 36883097.85 | 33815453.5 | 32941588.41 | 32540876.39 |
| Com_673_neg | LPC 19:1 | C27 H54 N O7 P | 581.36974 | 10.035 | 36596377.23 | 28144114.48 | 35141407.73 | 16810368.36 | 19147439.2 | 26197974.39 | 35366765.98 | 21540652.06 | 34385550.04 |
| Com_683_neg | N-Isobutyrylglycine | C6 H11 N O3 | 145.07385 | 4.807 | 18930884.12 | 18054541.07 | 16805520.25 | 21172786.45 | 15298065.44 | 46907061.22 | 22645379.05 | 24618773.68 | 24194309.87 |
| Com_687_neg | LPC 17:1 | C25 H50 N O7 P | 553.33864 | 9.629 | 31509948.76 | 24688590.82 | 29622563.02 | 23111925.64 | 25628653.37 | 32710052.7 | 37407303.35 | 29229774.8 | 29392615.1 |
| Com_722_neg | alpha-Ketoglutaric acid | C5 H6 O5 | 146.02147 | 1.621 | 15823858.81 | 29022215.4 | 35822941.98 | 28075049.2 | 25409533.64 | 25232030.8 | 22666280.35 | 24573711.63 | 23519619.68 |
| Com_737_neg | trans-10-Heptadecenoic Acid | C17 H32 O2 | 268.24014 | 10.183 | 27476278.36 | 29669872.97 | 25110984.82 | 35213871.09 | 27064826.92 | 27552008.94 | 34911137.46 | 31046383.63 | 32716120.1 |
| Com_761_neg | FAHFA (14:0/6:0) | C20 H38 O4 | 342.27692 | 8.044 | 22460853.49 | 33395254.34 | 24956299.28 | 18303850.92 | 16818918.29 | 22823770.36 | 25200511.72 | 19811493.41 | 27989948.08 |
| Com_767_neg | N-Acetyl-DL-phenylalanine | C11 H13 N O3 | 207.08946 | 5.72 | 32251856.51 | 27851229.78 | 25530433.24 | 26358374.24 | 29434225.37 | 29643654.97 | 21829261.09 | 25611956.34 | 34361842.09 |
| Com_769_neg | SM (d14:1/21:0) | C40 H81 N2 O6 P | 762.58984 | 11.071 | 26794632.48 | 21847546.63 | 26220912.01 | 21672670.08 | 20909814.46 | 41708866.48 | 24247753.44 | 20433393.9 | 16233665.82 |
| Com_787_neg | Reserpine | C33 H40 N2 O9 | 608.27779 | 3.409 | 6564005.223 | 31759748.81 | 4247793.717 | 14501563.8 | 23451692.95 | 13106302.03 | 7941597.634 | 11131908.95 | 8077317.935 |
| Com_796_neg | 11(Z)-Eicosenoic acid | C20 H38 O2 | 356.29272 | 9.846 | 30712766.05 | 27508932.32 | 26425631.26 | 26383126.3 | 28295991.12 | 26228230.31 | 26085957.83 | 23277625.91 | 23050882.26 |
| Com_798_neg | Butylparaben | C11 H14 O3 | 194.09389 | 6.017 | 16514608.76 | 19863202.96 | 16760068.64 | 26426815.29 | 29728459.94 | 39780193.67 | 21688132.56 | 23398677.44 | 23792172.46 |
| Com_801_neg | L-Ascorbic acid 2-sulfate | C6 H8 O9 S | 255.98887 | 1.439 | 25858989.33 | 31169224.47 | 19164316.01 | 20678055.1 | 23284625.61 | 30326657.38 | 30111536.26 | 26075241.72 | 26099676.14 |
| Com_805_neg | Prostaglandin D1 | C20 H34 O5 | 336.23006 | 6.892 | 24015098.93 | 21955208.65 | 27593732.75 | 26158054.18 | 31078378.99 | 39344381.34 | 27439958.31 | 24170639.91 | 27613943.25 |
| Com_831_neg | N-Acetyl-L-leucine | C8 H15 N O3 | 173.10518 | 5.687 | 21691483.38 | 29669494.23 | 18331058.34 | 25657736 | 29872533.52 | 33948144.64 | 23662225.1 | 24348631.29 | 26638173.65 |
| Com_851_neg | LPC 15:1 | C23 H46 N O7 P | 525.30689 | 8.625 | 20139956.12 | 24467909.3 | 24386016.78 | 21015395.53 | 22390403.21 | 33051503.97 | 29021387.25 | 27254930.81 | 24869915.42 |
| Com_854_neg | Ornithine | C5 H12 N2 O2 | 132.08982 | 1.352 | 18380569.62 | 17958721.85 | 12619089.06 | 15868477.51 | 33969669.12 | 31187921.55 | 23402481.93 | 25126413.99 | 18634191.13 |
| Com_855_neg | PE (18:0e/18:2) | C41 H80 N O7 P | 729.56881 | 11.256 | 27178208.72 | 7137709.089 | 3750114.908 | 2030809.789 | 33656063.04 | 4573739.61 | 20565176.61 | 30069192.51 | 24148844.7 |
| Com_858_neg | Lauric Acid | C12 H24 O2 | 200.17765 | 8.712 | 20195192.51 | 19092981.65 | 25481985.46 | 27342536.25 | 32675349.86 | 36130542.14 | 23580762.6 | 23571245.06 | 22049574.71 |
| Com_880_neg | LPC 19:0 | C27 H56 N O7 P | 583.38548 | 10.62 | 19109183.49 | 20582446.12 | 19939654.67 | 12547890.81 | 17714843.69 | 19602937.26 | 28266565.56 | 18329345.74 | 18434298.78 |
| Com_909_neg | (R)-3-Hydroxy myristic acid | C14 H28 O3 | 244.20373 | 7.899 | 26163948.12 | 19770780.34 | 17329518.48 | 26323614.44 | 18956051.1 | 24614970.66 | 21771320.32 | 20334247.13 | 21172104.52 |
| Com_942_neg | trans-Petroselinic acid | C18 H34 O2 | 346.27178 | 7.65 | 18774354.2 | 10543711.98 | 20595444.25 | 20151415.07 | 13112249.25 | 32473151.76 | 17067612.2 | 17120798.59 | 17941887.07 |
| Com_983_neg | PE (14:1e/18:1) | C37 H72 N O7 P | 673.50547 | 11.229 | 12961393.79 | 18568024.1 | 11051215.71 | 9615623.866 | 16911346.03 | 30854874.96 | 12622925.03 | 16441889.64 | 10578859.53 |
| Com_990_neg | FAHFA (18:1/20:3) | C38 H66 O4 | 586.49418 | 10.571 | 14022032.65 | 18710781.39 | 11605791.35 | 21984228.35 | 17829387.58 | 15826168.25 | 24312595.08 | 16858041.77 | 17505915.05 |
| Com_1018_neg | Xanthohumol | C21 H22 O5 | 354.14951 | 9.782 | 7062375.435 | 13186526.61 | 11028169.05 | 26250558.42 | 17444701.81 | 13804716.3 | 11068326.06 | 14848052.76 | 18162204.39 |
| Com_1020_neg | Glutaric Acid | C5 H8 O4 | 132.04217 | 1.313 | 19827831.46 | 21558881.75 | 19338106.33 | 26164277.59 | 25039616.12 | 21336846.76 | 21143770.21 | 23230334.35 | 22800182.02 |
| Com_1024_neg | Docosanoic Acid | C22 H44 O2 | 340.33405 | 10.923 | 20246978.25 | 16162556.76 | 21936904.16 | 14420670.97 | 27137055.48 | 14084661.57 | 17464362.94 | 13473549.85 | 15147700.79 |
| Com_1041_neg | 23-Norcholic acid | C23 H38 O5 | 394.27215 | 7.053 | 10684535.21 | 10605019.71 | 8684591.5 | 4773086.936 | 26717224.01 | 13599573.89 | 11462661.21 | 11841695.24 | 11761253.22 |
| Com_1053_neg | PE (14:1e/20:4) | C39 H70 N O7 P | 695.49022 | 11.707 | 12803344.36 | 9531696.072 | 13952933 | 8922851.051 | 26286481.59 | 13094993.05 | 12133827.86 | 12977894.68 | 11289045.35 |
| Com_1066_neg | LPE 22:5 | C27 H46 N O7 P | 527.30152 | 9.224 | 11786886.51 | 14351710.1 | 12026575.91 | 24735829.08 | 9229381.491 | 11015066.27 | 18693368.71 | 14302267.18 | 14992011.44 |
| Com_1074_neg | S-Lactoyglutathione | C13 H21 N3 O8 S | 379.10344 | 2.269 | 16917815.3 | 17620746.18 | 16550799.39 | 17900663.46 | 25426374.09 | 12260162.31 | 18105661.42 | 16867340.68 | 13119150.13 |
| Com_1145_neg | Erucic acid | C22 H42 O2 | 338.31858 | 9.125 | 12349435.03 | 6169976.46 | 16719125.54 | 8729310.56 | 23466903.57 | 11470210.65 | 12854509.23 | 9613439.636 | 9890191.751 |
| Com_1207_neg | 2,5-Dimethylphenol | C8 H10 O | 122.07298 | 11.977 | 18114830.75 | 15974580.44 | 14355824.93 | 11981299.59 | 16017160.53 | 13980954.16 | 11697901.27 | 13594981.23 | 15242340.01 |
| Com_1229_neg | 2,3-Dinor-8-epi-prostaglandin F2α | C18 H30 O5 | 652.41942 | 6.617 | 11470252.34 | 8237194.258 | 18261813.5 | 14406446.34 | 11805331.97 | 8673456.623 | 5378228.772 | 9987546.844 | 10348800.3 |
| Com_1233_neg | N-Oleoyl Dopamine | C26 H43 N O3 | 463.33017 | 7.866 | 9514049.22 | 12962321 | 6882625.441 | 7788747 | 5068180.012 | 22883299.48 | 11546464.32 | 9788799.936 | 7076191.327 |
| Com_1244_neg | 2-(acetylamino)-3-(1H-indol-3-yl)propanoic acid | C13 H14 N2 O3 | 246.10028 | 5.667 | 6907926.716 | 6368649.614 | 5718367.955 | 3590373.404 | 21204861.09 | 8758372.516 | 7522532.186 | 7098819.232 | 8301735.873 |
| Com_1266_neg | 11-Epiprostaglandin E1 | C20 H34 O5 | 336.22779 | 8.709 | 15052103.83 | 12556313.41 | 17528949.29 | 19673511.13 | 20038296.5 | 17246786.9 | 16601199.9 | 16624709.86 | 14908314.37 |
| Com_1280_neg | 4-Hydroxyisoleucine | C6 H13 N O3 | 147.0896 | 1.574 | 13891553.92 | 14264978.46 | 17342637.08 | 15926695.06 | 17550571.05 | 16743611.42 | 15403330.06 | 15035826.29 | 15534690.12 |
| Com_1281_neg | 17-AAG | C31 H43 N3 O8 | 567.28459 | 11.959 | 12879044.75 | 14100708.55 | 13726202.07 | 11281292.87 | 20396415.27 | 2381304.671 | 14697302.93 | 9696602.017 | 8904278.189 |
| Com_1291_neg | Hydroxyglutaric acid | C5 H8 O5 | 148.03708 | 2.258 | 15077320.37 | 17047014.02 | 16376108.71 | 7033890.046 | 14297715.77 | 7136732.692 | 14723029.18 | 14514011.94 | 10993254.53 |
| Com_1303_neg | 4-Acetamidobutyric Acid | C6 H11 N O3 | 145.07385 | 4.635 | 7638278.492 | 8623625.683 | 7310797.863 | 8806463.243 | 8776985.494 | 21419261.06 | 9821087.715 | 10728487.79 | 10889552.23 |
| Com_1340_neg | 23-Nordeoxycholic acid | C23 H38 O4 | 378.27699 | 9.056 | 12024682.81 | 14678654.18 | 16655608.83 | 12432445.29 | 15285129.71 | 20773458.29 | 16361697.87 | 13724071.17 | 11964900.61 |
| Com_1361_neg | LPC 20:1 | C28 H56 N O7 P | 595.38497 | 10.437 | 9811781.551 | 10614764.93 | 12248814.85 | 5476616.866 | 8559420.611 | 8208355.961 | 16460429.4 | 8655918.505 | 11317097.32 |
| Com_1375_neg | FAHFA (16:0/18:2) | C34 H62 O4 | 534.46275 | 10.382 | 12308199.49 | 14604501.64 | 8036594.636 | 16615170.21 | 15003544.88 | 18372733.6 | 16270352.78 | 13058347.36 | 14622061.33 |
| Com_1394_neg | Royal jelly acid | C10 H18 O3 | 186.12578 | 6.854 | 12461418.85 | 10359976.04 | 10385577.74 | 14351938.77 | 18883520.68 | 18757549.8 | 14637537.26 | 13118824.14 | 12926456.86 |
| Com_1395_neg | Lignoceric acid | C24 H48 O2 | 414.37103 | 10.415 | 13876016 | 13541867.77 | 11708688 | 16467875.11 | 14413362.68 | 14519896.97 | 16091797.56 | 15486716.74 | 15010323.89 |
| Com_1398_neg | 10-Hydroxydecanoic acid | C10 H20 O3 | 188.14087 | 7.165 | 12759451.77 | 12619650.86 | 12581987.83 | 18032745.9 | 14888126.04 | 17212618.83 | 13739626.3 | 14193431.59 | 13857095.4 |
| Com_1411_neg | N-Propionylglycine | C5 H9 N O3 | 131.0582 | 1.36 | 8756851.28 | 15683253.89 | 8274487.755 | 6270310.877 | 11361605.74 | 13124465.57 | 11011846.51 | 13025423.38 | 11639684.39 |
| Com_1413_neg | Citraconic acid | C5 H6 O4 | 130.02659 | 2.255 | 12627522.68 | 14902706.88 | 15803549.16 | 5755560.123 | 12184044.68 | 6680120.23 | 12780064.36 | 12792540.85 | 10274176.82 |
| Com_1446_neg | Glycerol 1-hexadecanoate | C19 H38 O4 | 330.277 | 11.183 | 8971874.547 | 15248740.63 | 7806475.894 | 13314770.76 | 11058702.65 | 10715082.62 | 11583248.12 | 12152728.82 | 15481592.53 |
| Com_1447_neg | Δ17-6-keto prostaglandin F1α | C20 H32 O6 | 414.22562 | 6.679 | 10995031.86 | 7109295.36 | 15380185.43 | 14634521.9 | 16138832.18 | 15189878.17 | 11939345.08 | 13660885.2 | 10910321.84 |
| Com_1453_neg | 2-Methylpentanedioic acid | C6 H10 O4 | 146.05806 | 5.054 | 14782897.21 | 7905294.493 | 10160710.79 | 8074056.986 | 6838731.653 | 11958497.24 | 9777445.554 | 10128362.96 | 9264712.955 |
| Com_1454_neg | Cer-NS (d18:2/16:0) | C34 H65 N O3 | 581.50252 | 11.336 | 12524754.61 | 14961158.96 | 14400103.92 | 10699305.52 | 13895890.83 | 17351693.19 | 15322020.65 | 11215985.44 | 14237283.67 |
| Com_1515_neg | Prostaglandin B2 | C20 H30 O4 | 334.21458 | 7.988 | 10394779.81 | 13350893.42 | 13011147.13 | 16319376.11 | 16101452.76 | 16603289.54 | 14379800.29 | 12712749.09 | 14014805.62 |
| Com_1532_neg | SM (d14:1/16:0) | C35 H71 N2 O6 P | 692.5115 | 8.943 | 8496012.241 | 11528760.54 | 6448652.695 | 7487265.621 | 9288252.491 | 6736773.855 | 12140387.87 | 8546121.838 | 15732307.33 |
| Com_1561_neg | PE (14:1e/18:2) | C37 H70 N O7 P | 671.48897 | 9.458 | 7525171.764 | 9049538.443 | 7268016.915 | 11279552.13 | 8010547.875 | 17549522.16 | 7468895.155 | 7991828.586 | 8661500.512 |
| Com_1574_neg | Erythronolactone | C4 H6 O4 | 118.02651 | 2.194 | 6843254.604 | 4893014.725 | 13884132.9 | 6755497.543 | 2035195.005 | 4815793.295 | 5413958.482 | 5608875.532 | 5464735.103 |
| Com_1575_neg | PE (16:2e/20:4) | C41 H72 N O7 P | 721.50524 | 10.305 | 6173802.347 | 4268812.876 | 4795810.504 | 5340621.347 | 6694712.718 | 17397326.93 | 5366469.889 | 6293437.611 | 3531755.842 |
| Com_1591_neg | Homoarginine | C7 H16 N4 O2 | 188.11609 | 1.591 | 13227027.99 | 10487231.91 | 5494468.463 | 13029193.05 | 13518691.06 | 8722890.694 | 10386501.81 | 10917828.26 | 10813572.17 |
| Com_1605_neg | Oleoyl-L-α-lysophosphatidic acid | C21 H41 O7 P | 436.25917 | 9.434 | 6847579.097 | 13405822.28 | 10624904.31 | 14624800.91 | 14296988.56 | 12375869.35 | 11434618.82 | 12677918.39 | 13210107.8 |
| Com_1643_neg | Prostaglandin H1 | C20 H34 O5 | 336.23007 | 8.279 | 12701772.31 | 8473277.822 | 9746425.843 | 7485409.319 | 9131347.962 | 8706149.454 | 8422832.034 | 7594351.706 | 7737478 |
| Com_1646_neg | Dimetghyl 4-Hydroxyisophthalate | C10 H10 O5 | 210.05284 | 5.598 | 7777538.222 | 10377496.36 | 10253690.16 | 8123840.783 | 15402684.49 | 16130761.22 | 9708440.015 | 9483902.323 | 11620079.73 |
| Com_1674_neg | 5-Aminopentanoate | C5 H11 N O2 | 117.079 | 5.154 | 10669067.59 | 4453824.671 | 7641015.068 | 10809175.64 | 15029394.33 | 4359931.596 | 7267668.844 | 6885164.016 | 6536828.552 |
| Com_1687_neg | 13,14-dihydro-15-keto-tetranor Prostaglandin D2 | C16 H26 O5 | 280.16505 | 7.172 | 12284546.88 | 9093768.616 | 12596770.96 | 11696286.81 | 5344843.032 | 11398330.43 | 10348867.44 | 11497513 | 10795432.03 |
| Com_1690_neg | Paracetamol | C8 H9 N O2 | 151.0633 | 5.103 | 12270956.21 | 5736424.078 | 7533518.359 | 9887744.62 | 7265104.362 | 10933599.08 | 8874572.446 | 9381453.833 | 8959285.502 |
| Com_1734_neg | FAHFA (18:2/20:4) | C38 H62 O4 | 582.46333 | 10.042 | 7742477.072 | 8209781.096 | 5983027.922 | 9701703.103 | 10258541.2 | 9548597.426 | 12311791.15 | 8403313.576 | 9467066.229 |
| Com_1740_neg | PE (18:0/20:4) | C43 H78 N O8 P | 767.54532 | 11.231 | 7692480.13 | 12106966.09 | 6133586.944 | 6994144.538 | 13516668.89 | 10022822.22 | 7573417.652 | 7183438.581 | 11708890.89 |
| Com_1751_neg | PC (5:0/13:1) | C26 H50 N O8 P | 581.33343 | 7.426 | 6368816.418 | 10410131.05 | 7523537.468 | 9510447.711 | 10312338.63 | 12751776.12 | 12180780.41 | 9996652.126 | 10662769.42 |
| Com_1754_neg | Corey Lactone Diol | C8 H12 O4 | 172.07355 | 5.61 | 11741185.07 | 7787152.662 | 9082861.307 | 10561114.13 | 9268184.116 | 11156115.95 | 8704018.508 | 8738208.184 | 11235086.71 |
| Com_1758_neg | Nonadecanoic acid | C19 H38 O2 | 298.28702 | 11.004 | 11712467.44 | 11271382.92 | 8614001.479 | 11457178.84 | 7497910.67 | 10139353.36 | 11965570.87 | 9776220.412 | 10557764.38 |
| Com_1759_neg | Ethyl-β-D-glucuronide | C8 H14 O7 | 222.07395 | 2.135 | 10911537.42 | 3685207.833 | 12100992.73 | 13499203.64 | 2677336.205 | 5238418.765 | 6442060.928 | 6750695.278 | 6681033.367 |
| Com_1773_neg | 2-Phenylpropionic acid | C9 H10 O2 | 150.06796 | 6.206 | 8815424.491 | 10613578.92 | 11603636.51 | 12730680.5 | 10575435.44 | 14827954.43 | 11995461.97 | 9285404.014 | 7975021.553 |
| Com_1806_neg | cis-Aconitic acid | C6 H6 O6 | 174.01642 | 1.45 | 1961864.595 | 11476670.48 | 1449744.651 | 1091573.432 | 1557478.101 | 8837213.805 | 1599287.54 | 1199851.725 | 1240109.98 |
| Com_1816_neg | Kynurenine | C10 H12 N2 O3 | 208.08477 | 4.684 | 6931315.334 | 7910659.803 | 8818276.946 | 7368503.36 | 6605883.786 | 14465755.4 | 8249238.683 | 8434300.409 | 8192820.198 |
| Com_1824_neg | Methionine | C5 H11 N O2 S | 149.05091 | 2.033 | 3684120.82 | 9508799.36 | 5771176.597 | 6297023.995 | 13469600.24 | 7621503.538 | 6855844.598 | 4673023.344 | 6319924.689 |
| Com_1827_neg | trans-Cinnamic acid | C9 H8 O2 | 148.05246 | 4.916 | 7436430.799 | 9456275.69 | 7055671.176 | 12588546.31 | 12767207.15 | 14327619.71 | 8583157.381 | 9488462.576 | 10614176.95 |
| Com_1844_neg | Epinephrine | C9 H13 N O3 | 183.08945 | 5.644 | 5767019.411 | 4109911.952 | 6761177.641 | 12770008.51 | 4564301.085 | 6165679.638 | 6613496.461 | 7059609.912 | 6391561.738 |
| Com_1852_neg | 1,2,3,9-tetrahydro-4H-carbazol-4-one oxime | C12 H12 N2 O | 200.09497 | 5.09 | 4062848.132 | 11150860.97 | 5488234.041 | 3749779.625 | 9403810.311 | 8043789.769 | 5507654.528 | 5319922.814 | 5409226.918 |
| Com_1878_neg | D-Threose | C4 H8 O4 | 120.04221 | 1.312 | 8682171.251 | 10137831.1 | 8551440.508 | 11688317.4 | 11268970.16 | 10592710.8 | 11166791.92 | 11231251.99 | 10907460.27 |
| Com_1915_neg | Docosatrienoic acid | C22 H38 O2 | 334.28727 | 11.046 | 7001969.337 | 10709150.88 | 6661533.767 | 8131037.305 | 6984344.947 | 5944493.317 | 10480080.17 | 9019798.259 | 8133991.438 |
| Com_1917_neg | Cer-NS (d16:1/16:0) | C32 H63 N O3 | 555.48652 | 11.065 | 4806872.954 | 9796466.018 | 5302104.959 | 5360247.02 | 11191422.62 | 7768589.374 | 6938505.172 | 6973773.87 | 11963276.23 |
| Com_1921_neg | 19(R)-hydroxy Prostaglandin A2 | C20 H30 O5 | 332.19885 | 7.189 | 6374699.667 | 8223757.936 | 4609894.909 | 6151301.955 | 12379621.62 | 13526268.77 | 8689660.373 | 7841873.706 | 7339984.885 |
| Com_1938_neg | L-(+)-Arabinose | C5 H10 O5 | 150.05277 | 1.413 | 10287163 | 8282355.025 | 4889030.581 | 7362165.643 | 8871212.4 | 7465570.189 | 10695872.98 | 9518593.418 | 8778604.907 |
| Com_1941_neg | trans-Aconitic acid | C6 H6 O6 | 174.01631 | 2.054 | 9088589.466 | 7983408.68 | 10615382.35 | 4065914.383 | 6074678.756 | 4627834.083 | 7036473.108 | 7072075.943 | 6762456.484 |
| Com_1981_neg | Cer-NS (d17:1/16:0) | C33 H65 N O3 | 569.50246 | 11.732 | 7907446.262 | 10273486.93 | 8605043.537 | 8382939.616 | 11483811.97 | 11905280.43 | 8916458.765 | 8482063.006 | 10965766.41 |
| Com_1988_neg | 12-epi Leukotriene B4 | C20 H32 O4 | 354.24061 | 7.822 | 5522857.102 | 9054183.299 | 7389900.328 | 5111189.098 | 9968435.02 | 12584115.5 | 10356660.42 | 10725857.78 | 8880077.503 |
| Com_1990_neg | o-Toluic Acid | C8 H8 O2 | 136.05235 | 6.272 | 6030669.426 | 5593008.068 | 6361989.615 | 7524455.053 | 9932792.1 | 12923631.12 | 6342912.694 | 5866211.764 | 6927232.722 |
| Com_1996_neg | 3'-Adenosine monophosphate (3'-AMP) | C10 H14 N5 O7 P | 347.0616 | 5.14 | 6797842.182 | 10177628.18 | 6156100.896 | 6053573.276 | 8683230.008 | 9602206.194 | 7876264.682 | 8250482.133 | 7512273.112 |
| Com_2006_neg | (±)8(9)-DiHET | C20 H34 O4 | 338.24337 | 10.746 | 6386688.052 | 7335037.061 | 5232031.597 | 11435675.06 | 8039284.731 | 8723521.824 | 8382548.273 | 7170914.87 | 8996598.749 |
| Com_2010_neg | (±)9(10)-EpOME | C18 H32 O3 | 342.24054 | 8.211 | 5765014.443 | 9560643.166 | 7416926.688 | 8538989.547 | 11376193.12 | 8999271.268 | 10135429.73 | 8170147.323 | 9128295.694 |
| Com_2016_neg | 2-Arachidonyl glycerol ether | C23 H40 O3 | 410.30296 | 9.579 | 9074238.894 | 8743471.793 | 8960431.566 | 7704692.542 | 9330458.035 | 9920479.039 | 10078657.32 | 8725188.609 | 8452650.466 |
| Com_2017_neg | L-Ascorbate | C6 H8 O6 | 176.03192 | 5.624 | 9719241.087 | 4289502.807 | 2741857.256 | 2885473.269 | 2725360.542 | 3241626.742 | 3791523.502 | 3948727.556 | 4945254.852 |
| Com_2040_neg | glutathione disulfide | C20 H32 N6 O12 S2 | 612.15194 | 6.706 | 9548395.634 | 7702250.852 | 6051952.949 | 4585105.918 | 6392210.454 | 5579990.597 | 3234341.936 | 4651922.295 | 2831187.873 |
| Com_2132_neg | 12-Epileukotriene B4 | C20 H32 O4 | 336.23009 | 7.816 | 5149796.454 | 8379927.461 | 6653470.36 | 4491152.741 | 9910962.523 | 11084018.8 | 9265018.824 | 7016748.02 | 7925408.496 |
| Com_2147_neg | PC(16:0/16:0) | C40 H80 N O8 P | 733.56337 | 9.783 | 356238.6157 | 9010415.589 | 334833.4874 | 606687.4699 | 622927.4777 | 495102.3305 | 1007019.017 | 563337.8824 | 754367.015 |
| Com_2181_neg | NSI-189 | C22 H30 N4 O | 366.24048 | 8.293 | 7367132.69 | 4779499.442 | 6998413.955 | 6131964.376 | 6482348.496 | 11180951.68 | 7313280.244 | 6971491.997 | 5511263.158 |
| Com_2185_neg | trans-2-Butene-1,4-dicarboxylic Acid | C6 H8 O4 | 144.0422 | 1.309 | 7229154.584 | 8402685.046 | 7308053.895 | 9656336.284 | 8696851.711 | 7615382.63 | 8519204.908 | 8179465.063 | 9853037.306 |
| Com_2228_neg | Pentadecanoic Acid | C15 H30 O2 | 288.23001 | 7.457 | 5146719.687 | 5700610.237 | 6175846.467 | 8296810.962 | 4858553.735 | 10793445.63 | 8618274.09 | 7239223.024 | 8302328.104 |
| Com_2230_neg | Porphobilinogen | C10 H14 N2 O4 | 226.09545 | 4.903 | 8319203.898 | 7966628.167 | 7608501.634 | 9271306.536 | 8418450.476 | 8686365.97 | 8077407.357 | 8199820.118 | 8498991.631 |
| Com_2269_neg | LPE 16:1 | C21 H42 N O7 P | 451.27014 | 8.811 | 6893638.358 | 7472248.019 | 6547262.349 | 8395100.861 | 5058408.678 | 5542211.38 | 8398117.913 | 7115493.339 | 7217589.23 |
| Com_2298_neg | D-Phenylalanine | C9 H11 N O2 | 165.07884 | 5.592 | 7972053.939 | 4133007.124 | 7420180.113 | 7345674.087 | 7683872.925 | 4585837.468 | 6430277.807 | 5596584.342 | 5581250.758 |
| Com_2300_neg | Arginine | C6 H14 N4 O2 | 174.11154 | 1.305 | 5258131.024 | 7614552.991 | 8233360.817 | 8096479.487 | 8368902.756 | 7745276.607 | 6832322.532 | 7513756.526 | 8289013.449 |
| Com_2301_neg | SM (d14:2/18:0) | C37 H73 N2 O6 P | 718.52703 | 9.608 | 6008123.776 | 6166784.477 | 6556248.411 | 5810604.656 | 6475878.044 | 6575059.022 | 8255683.656 | 4640927.682 | 6823769.562 |
| Com_2330_neg | LPC 20:0 | C28 H58 N O7 P | 597.40143 | 11.011 | 5946200.944 | 6404282.591 | 5514516.225 | 4176963.356 | 5785205.08 | 6298115.701 | 8132000.62 | 5432780.61 | 5540682.606 |
| Com_2340_neg | Mevalonic acid | C6 H12 O4 | 148.07349 | 3.993 | 7813009.893 | 3555126.79 | 7768317.129 | 6418854.719 | 1880489.281 | 3174582.512 | 4152605.449 | 4242493.285 | 4662212.897 |
| Com_2355_neg | 2-(3,4-dimethoxyphenyl)ethanamine | C10 H15 N O2 | 181.11023 | 6.626 | 7731011.504 | 4569920.725 | 4409587.637 | 5551974.585 | 6348666.638 | 8006630.377 | 5484998.446 | 5681663.845 | 5361450.047 |
| Com_2392_neg | Pyrogallol | C6 H6 O3 | 126.03151 | 4.814 | 7577136.448 | 4547010.246 | 6791870.078 | 6519483.85 | 2913142.588 | 4541128.714 | 6184044.625 | 5331362.394 | 5335613.998 |
| Com_2406_neg | 15(R),19(R)-Hydroxy prostaglandin F1α | C20 H36 O6 | 354.24083 | 6.698 | 6116010.253 | 7318634.825 | 5797821.574 | 6306854.661 | 6620234.43 | 9757308.1 | 6355184.993 | 4925850.619 | 7128911.095 |
| Com_2461_neg | LPC 14:1 | C22 H44 N O7 P | 511.29119 | 8.055 | 3943160.709 | 6382890.024 | 5663043.695 | 4236749.559 | 5627000.364 | 8366864.137 | 7261920.108 | 7792545.063 | 5604673.326 |
| Com_2462_neg | LPC 19:2 | C27 H52 N O7 P | 579.35416 | 9.53 | 6653679.95 | 6198503.077 | 7474582.717 | 2957714.131 | 5843140.122 | 5534909.47 | 6364933.799 | 5522475.553 | 4939311.454 |
| Com_2510_neg | 1-methyl-N-(3-methyl-5-cinnolinyl)-1H-imidazole-4-sulfonamide | C13 H13 N5 O2 S | 303.07756 | 5.799 | 7019749.464 | 3909297.24 | 4266360.857 | 2579706.608 | 1558609.393 | 5348391.563 | 3534730.935 | 3765411.498 | 3610625.133 |
| Com_2610_neg | 4-Oxoretinol | C20 H28 O2 | 300.20885 | 9.151 | 4097674.254 | 3475476.563 | 5227182.817 | 7721409.562 | 5403278.414 | 5987067.358 | 5781573.364 | 5420935.523 | 5390798.32 |
| Com_2634_neg | LPE 16:0 | C21 H44 N O7 P | 453.28573 | 9.188 | 5266485.385 | 5775197.475 | 5266815.026 | 6897448.725 | 5553584.928 | 6895744.018 | 6761984.465 | 5243920.758 | 6131453.48 |
| Com_2635_neg | PC (15:0/18:2) | C41 H78 N O8 P | 789.55263 | 10.323 | 3838215.199 | 3837727.46 | 4849629.036 | 4050913.589 | 7046274.848 | 6235584.043 | 6761600.218 | 3650525.555 | 4480012.093 |
| Com_2667_neg | PC (14:1e/18:1) | C40 H78 N O7 P | 761.5594 | 11.447 | 5825253.813 | 4305649.598 | 3766240.059 | 5019232.859 | 3934517.288 | 8297161.169 | 5293150.344 | 5323425.753 | 3834800.148 |
| Com_2701_neg | 10-Undecenoic acid | C11 H20 O2 | 184.1465 | 6.536 | 1640558.732 | 2216636.53 | 5583541.741 | 6469193.263 | 3410689.732 | 8136141.766 | 3687417.453 | 4124784.833 | 3987082.09 |
| Com_2710_neg | acetoacetate | C4 H6 O3 | 102.03162 | 1.31 | 4794922.628 | 5924012.39 | 5247451.165 | 6738657.553 | 6529063.193 | 6350452.677 | 6480149.693 | 6158265.326 | 6393783.053 |
| Com_2726_neg | PE (16:0/20:5) | C41 H72 N O8 P | 737.50084 | 11.778 | 4804320.921 | 5113986.83 | 6420152.378 | 3808523.035 | 4823114.597 | 1645565.281 | 4641654.702 | 4349296.842 | 4669068.509 |
| Com_2742_neg | Lysopc 16:1 | C24 H48 N O7 P | 493.31664 | 9.249 | 4580259.985 | 5048526.623 | 6358943.406 | 4231649.466 | 2353745.184 | 5005428.37 | 5800592.006 | 4402867.234 | 6380335.527 |
| Com_2744_neg | (3R)-4,4-Dimethyl-2-oxotetrahydro-3-furanyl beta-D-glucopyranoside | C12 H20 O8 | 338.12138 | 4.911 | 6147867.426 | 3829748.321 | 3536365.967 | 5104696.982 | 4154347.319 | 4830890.095 | 4271305.188 | 3930847.098 | 3697033.388 |
| Com_2749_neg | Allolithocholic acid | C24 H40 O3 | 376.29774 | 9.014 | 4152459.208 | 3485341.048 | 6321505.969 | 4949001.786 | 2961524.056 | 3802006.065 | 4845049.597 | 3960659.873 | 4773144.792 |
| Com_2763_neg | SM (d14:2/19:0) | C38 H75 N2 O6 P | 732.54345 | 11.092 | 3898188.633 | 6178839.853 | 4212895.813 | 3686575.164 | 3808360.949 | 4664861.444 | 5490524.879 | 2632079.925 | 4621457.132 |
| Com_2783_neg | PC (16:1e/18:2) | C42 H80 N O7 P | 787.57401 | 11.951 | 4982645.028 | 1983114.963 | 6193156.811 | 4427282.393 | 2896095.04 | 6970801.857 | 3817396.614 | 4432769.582 | 3231698.695 |
| Com_2811_neg | SM (d14:3/18:1) | C37 H69 N2 O6 P | 714.49609 | 11.885 | 5060337.003 | 6065382.862 | 3065609.733 | 3327307.458 | 3351842.032 | 3284032.105 | 5168977.269 | 3988403.429 | 3614533.989 |
| Com_2877_neg | Lysops 22:6 | C28 H44 N O9 P | 569.27344 | 9.617 | 3313749.194 | 4735766.101 | 2872192.137 | 4346286.724 | 6896798.957 | 5347640.986 | 3848500.806 | 6152104.421 | 5523257.946 |
| Com_2879_neg | Cer-EODS (d22:0/12:0-O-16:0) | C50 H99 N O5 | 793.75315 | 10.344 | 3678908.916 | 3353861.285 | 5411613.496 | 4535034.305 | 4700594.658 | 7401229.935 | 4134346.261 | 4762538.059 | 5429525.184 |
| Com_2900_neg | Nervonic acid | C24 H46 O2 | 366.34981 | 10.085 | 4446985.008 | 5449380.767 | 5017879.935 | 4897112.301 | 4759104.342 | 3810381.964 | 5861213.277 | 5258889.408 | 3510778.719 |
| Com_2983_neg | P-Coumaric acid | C9 H8 O3 | 164.04729 | 2.28 | 3532564.626 | 5530347.804 | 5087231.897 | 4216981.643 | 5694170.933 | 4075411.016 | 2518199.135 | 4412745.982 | 4515393.754 |
| Com_3060_neg | N2-(2-methoxyphenyl)-5-chloro-3-methylbenzo[b]thiophene-2-sulfonamide | C16 H14 Cl N O3 S2 | 367.01164 | 5.628 | 5176486.18 | 3037043.058 | 2243769.834 | 3000895.587 | 2798795.464 | 2664152.05 | 2766314.309 | 3318427.673 | 4146013.206 |
| Com_3083_neg | Gluconolactone | C6 H10 O6 | 178.04749 | 1.345 | 4146549.135 | 3179169.955 | 3657972.831 | 5703018.738 | 4863615.812 | 3814457.779 | 3155302.319 | 5504585.181 | 4238988.44 |
| Com_3088_neg | 2-Hydroxy-1-(4-methoxyphenyl)propyl hexopyranoside | C16 H24 O8 | 390.15268 | 5.935 | 4200891.08 | 2138158.002 | 4226721.043 | 5924553.917 | 2087947.469 | 3190191.594 | 4240024.56 | 4661417.463 | 3250685.133 |
| Com_3142_neg | Pyridoxine O-Glucoside | C14 H21 N O8 | 331.12682 | 5.26 | 1600168.491 | 2918967.424 | 1382336.354 | 1581170.299 | 988993.4956 | 6429648.258 | 2015794.073 | 1869724.582 | 3048589.65 |
| Com_3254_neg | 3-Phosphoglyceric acid | C3 H8 Na O6 P | 193.9958 | 1.358 | 3867151.271 | 3867964.89 | 2845578.816 | 3464786.217 | 5666705.509 | 4918556.854 | 3741078.169 | 4590129.275 | 4678560.059 |
| Com_3305_neg | Mag (18:1) | C21 H40 O4 | 356.29269 | 10.202 | 3347687.757 | 4579320.658 | 3449151.281 | 5288851.802 | 5103771.36 | 5126557.468 | 4039820.86 | 3805865.17 | 3949819.901 |
| Com_3311_neg | PC (17:0/18:2) | C43 H82 N O8 P | 817.584 | 11.954 | 3221092.779 | 1440560.029 | 4677391.858 | 4953317.298 | 1276261.373 | 4895382.977 | 1353345.951 | 1684099.636 | 1280485.15 |
| Com_3318_neg | 5-Methoxyindole-3-Carbaldehyde | C10 H9 N O2 | 175.06321 | 5.209 | 2255473.406 | 1983161.616 | 4663137.455 | 2898888.23 | 3892195.244 | 2202930.936 | 2660461.747 | 2950123.364 | 3113376.317 |
| Com_3342_neg | N7-Methylguanosine | C11 H17 N5 O5 | 299.12153 | 1.344 | 2408177.861 | 4561611.553 | 1571474.099 | 2332006.59 | 4425007.06 | 3024571.943 | 3588158.615 | 2623586.754 | 3212424.015 |
| Com_3385_neg | GW501516 | C21 H18 F3 N O3 S2 | 453.066 | 1.392 | 2282218.348 | 3109784.79 | 878561.8192 | 1304556.725 | 5314658.616 | 2744472.135 | 3686859.79 | 3137969.123 | 3091336.903 |
| Com_3402_neg | 2,3-Dinor-TXB2 | C18 H30 O6 | 342.20375 | 7.567 | 3350815.661 | 4422039.253 | 784918.1832 | 4981323.996 | 2817073.681 | 3079833.996 | 2528021.984 | 2767685.69 | 3186156.223 |
| Com_3432_neg | Kynurenic acid | C10 H7 N O3 | 189.04277 | 5.099 | 1861813.51 | 2769187.507 | 3540011.716 | 2090358.165 | 5174189.337 | 2956099.684 | 2792931.791 | 3304145.587 | 2872871.166 |
| Com_3453_neg | PC (16:2e/18:3) | C42 H76 N O7 P | 783.54257 | 11.925 | 3354836.949 | 3025196.345 | 4027826.252 | 2811334.529 | 4621242.736 | 3101114.667 | 4365536.739 | 2841582.746 | 3882777.052 |
| Com_3457_neg | PE (22:4e/20:4) | C47 H80 N O7 P | 801.55293 | 10.932 | 1503939.775 | 3204273.379 | 1497748.922 | 4895541.15 | 3139795.809 | 2536516.303 | 1607914.3 | 1651939.895 | 2343077.419 |
| Com_3502_neg | N-(5-Aminopentyl)acetamide | C7 H16 N2 O | 144.12629 | 5.024 | 354053.1194 | 589605.3303 | 4214167.211 | 513333.243 | 608011.7339 | 430756.6141 | 985907.3321 | 1193885.401 | 938913.9998 |
| Com_3524_neg | PE (14:1e/16:0) | C35 H70 N O7 P | 647.49049 | 10.724 | 1725195.68 | 1764182.935 | 2090590.319 | 2543801.563 | 2104737.425 | 5236595.936 | 1617986.109 | 2513847.625 | 1482619.002 |
| Com_3619_neg | 1-(2,4-dihydroxyphenyl)-2-(3,5-dimethoxyphenyl)propan-1-one | C17 H18 O5 | 320.12937 | 11.978 | 3865555.596 | 3822811.81 | 2574924.424 | 3212335.335 | 3899520.447 | 3297247.89 | 3140791.933 | 2557982.577 | 3139537.794 |
| Com_3668_neg | Prostaglandin A1 ethyl ester | C22 H36 O4 | 346.25077 | 8.042 | 2765550.298 | 3391308.149 | 2931232.889 | 3661192.459 | 3015437.911 | 4223892.497 | 3918143.946 | 3319859.828 | 3186437.707 |
| Com_3738_neg | L-Glutamic acid monosodium salt | C5 H8 N Na O4 | 169.03503 | 1.343 | 1883958.365 | 3753399.972 | 3011638.319 | 2915641.425 | 3460100.624 | 2745443.98 | 2721776.419 | 3265791.028 | 2551141.031 |
| Com_3845_neg | N-Acetyl-Asp-Glu | C11 H16 N2 O8 | 304.09055 | 4.8 | 3475918.274 | 1757187.063 | 2100778.166 | 2677380.865 | 2363309.123 | 2871324.356 | 2515270.005 | 2391406.314 | 2420884.258 |
| Com_3880_neg | Methyl 3-indolyacetate | C11 H11 N O2 | 189.07878 | 5.988 | 1007251.013 | 654807.9785 | 1506180.651 | 556995.6356 | 4170872.695 | 755707.5361 | 1155388.222 | 1317680.188 | 1028409.849 |
| Com_3892_neg | Xanthine | C5 H4 N4 O2 | 152.03328 | 2.129 | 764991.5803 | 3491364.158 | 202173.8085 | 618422.4662 | 944859.5608 | 1361441.168 | 1128259.052 | 1173038.399 | 1015196.075 |
| Com_3911_neg | Adenylocuccinic Acid | C14 H18 N5 O11 P | 463.07454 | 2.298 | 2417381.049 | 2694918.044 | 3378707.845 | 1952526.23 | 4127833.419 | 2681971.247 | 2290015.089 | 2334830.065 | 2723739.594 |
| Com_4038_neg | LPC 22:3 | C30 H56 N O7 P | 619.38564 | 10.143 | 2589438.71 | 3131304.997 | 2282789.554 | 1531584.182 | 1567278.629 | 1704570.909 | 3311840.785 | 1994199.61 | 1992910.018 |
| Com_4114_neg | (±)19(20)-DiHDPA | C22 H34 O4 | 362.24574 | 8.557 | 2401291.628 | 3163597.619 | 2417429.968 | 2460584.29 | 2884798.895 | 3015765.079 | 2897366.188 | 2501711.307 | 1896947.472 |
| Com_4143_neg | 5-Hydroxytryptophol | C10 H11 N O2 | 177.07901 | 5.899 | 3049360.704 | 2651516.799 | 1330476.708 | 1946438.106 | 2010767.417 | 2830312.353 | 2167763.48 | 2490232.317 | 2056486.479 |
| Com_4150_neg | Bisphenol A | C15 H16 O2 | 228.11503 | 5.984 | 1591980.025 | 1743490.133 | 2049932.272 | 2045170.51 | 3691741.479 | 3018055.501 | 2254838.543 | 2547230.753 | 2620588.37 |
| Com_4173_neg | Argininosuccinic acid | C10 H18 N4 O6 | 290.12688 | 5.036 | 2058529.008 | 1663752.754 | 3103125.214 | 1889789.487 | 3257322.04 | 2193465.272 | 2432231.464 | 2807315.885 | 2292236.048 |
| Com_4232_neg | N6-Succinyl Adenosine | C14 H17 N5 O8 | 383.10626 | 2.748 | 2365666.806 | 2989566.47 | 1930790.057 | 2956492.787 | 2499992.347 | 2093571.283 | 2780871.431 | 1928328.969 | 2784061.437 |
| Com_4298_neg | Orsellinic acid ethyl ester | C10 H12 O4 | 196.0735 | 5.615 | 1949246.26 | 1628463.256 | 2726211.142 | 2792674.291 | 2774214.57 | 2649610.605 | 2461985.381 | 3063709.498 | 2875717.323 |
| Com_4348_neg | Homogentisic acid | C8 H8 O4 | 168.04229 | 4.735 | 1004965.423 | 800322.7732 | 583550.5329 | 601581.8825 | 951149.1573 | 3610934.918 | 1063772.008 | 824226.0685 | 857317.8208 |
| Com_4404_neg | Heneicosanoic acid | C21 H42 O2 | 326.31826 | 9.89 | 1888512.634 | 1684726.481 | 1905129.722 | 942570.9047 | 2775770.298 | 1271653.828 | 2814251.97 | 1780946.691 | 1827511.007 |
| Com_4406_neg | 4-Hydroxy-3- methoxyphenylglycol sulfate | C9 H12 O7 S | 264.0303 | 2.489 | 2713018.233 | 1609398.41 | 2269485.706 | 1651665.913 | 2232042.438 | 1812934.666 | 1834188.933 | 2024668.144 | 2134954.711 |
| Com_4450_neg | APS | C10 H14 N5 O10 P S | 427.02143 | 2.049 | 2659226.846 | 2660806.395 | 1198207.391 | 2597561.856 | 2726869.612 | 2185162.317 | 2397036.693 | 1788506.842 | 2516278.889 |
| Com_4524_neg | Cer-EODS (d22:0/12:0-O-18:1) | C52 H101 N O5 | 819.77049 | 11.267 | 695625.9249 | 2462655.064 | 521521.8094 | 539004.2008 | 1367722.8 | 455947.2404 | 2658529.262 | 1593993.329 | 1746297.047 |
| Com_4529_neg | Cer-EODS (d20:0/12:0-O-18:0) | C50 H99 N O5 | 793.75312 | 11.134 | 2560892.454 | 1398240.958 | 1716581.138 | 1492882.829 | 1335141.553 | 2291636.974 | 1172085.121 | 882427.1264 | 1568942.707 |
| Com_4590_neg | Calcitriol | C27 H44 O3 | 416.32874 | 9.704 | 2497484.681 | 2538110.445 | 969763.5874 | 1199988.733 | 2772998.757 | 2204604.249 | 2383925.832 | 1818754.72 | 1913450.06 |
| Com_4610_neg | Purine | C5 H4 N4 | 120.04217 | 1.506 | 2472102.276 | 2082264.347 | 1789475.252 | 1856355.52 | 2211034.29 | 2125116.831 | 2101206.689 | 1731461.263 | 2034276.517 |
| Com_4854_neg | D-2-Aminoadipic acid | C6 H11 N O4 | 161.06874 | 2.012 | 2191519.865 | 2185190.578 | 2202623.214 | 2445590.741 | 2341115.381 | 2678964.535 | 2309794.998 | 2308095.712 | 2284429.51 |
| Com_4892_neg | Cyclocytidine | C9 H12 Cl N3 O4 | 261.05254 | 2.961 | 1707530.029 | 1557752.082 | 1490198.929 | 1823142.362 | 2653957.616 | 2206038.55 | 1877005.635 | 1928491.262 | 1825684.091 |
| Com_4991_neg | Hydrocortisone acetate | C23 H32 O6 | 404.22047 | 8.609 | 1482710.8 | 1332620.48 | 2146442.862 | 1988110.111 | 2101645.106 | 2438199.331 | 1869182.693 | 1694503.778 | 1381369.439 |
| Com_5033_neg | 4-Methylvaleric Acid | C6 H12 O2 | 116.08387 | 5.92 | 2001809.781 | 1895866.312 | 1445215.818 | 1523976.456 | 1490948.87 | 2379178.011 | 2107987.856 | 1692498.362 | 2038618.154 |
| Com_5135_neg | FAHFA (18:1/19:2) | C37 H66 O4 | 574.49412 | 10.651 | 1097716.582 | 1284951.479 | 801591.1955 | 2264781.693 | 853537.5536 | 979975.5342 | 1698784.345 | 1236487.073 | 1405117.187 |
| Com_5177_neg | L-Methionine sulfone | C5 H11 N O4 S | 181.04082 | 2.054 | 1398977.757 | 1838036.089 | 1421904.201 | 1569151.572 | 1773880.186 | 1201466.043 | 1968736.813 | 1448075.672 | 1508259.769 |
| Com_5203_neg | Ursolic acid | C30 H48 O3 | 456.3613 | 9.69 | 1477300.9 | 960953.5512 | 923018.4024 | 894746.9449 | 793011.1541 | 2437499.881 | 1054723.402 | 1052455.619 | 1006765.079 |
| Com_5405_neg | Capric acid | C10 H20 O2 | 172.14624 | 7.436 | 955254.6817 | 987193.1592 | 1403559.17 | 2019686.664 | 1136918.888 | 1498650.707 | 1739901.988 | 1288979.202 | 1270886.088 |
| Com_5526_neg | Leukotriene C4 | C30 H47 N3 O9 S | 625.30804 | 5.001 | 1306984.885 | 664560.3062 | 1010806.023 | 738869.9666 | 970640.1707 | 2116325.95 | 1215718.808 | 1178677.512 | 1019544.354 |
| Com_5685_neg | Pyridoxamine | C8 H12 N2 O2 | 168.08979 | 2.807 | 722487.8095 | 1038594.901 | 660058.0557 | 770560.0122 | 1831488.941 | 1307400.925 | 923421.9753 | 821278.3497 | 812104.2138 |
| Com_5694_neg | N-Acetyl-L-methionine | C7 H13 N O3 S | 191.06164 | 3.098 | 749707.7159 | 1536173.892 | 594147.0083 | 873414.9608 | 1215223.945 | 1064651.583 | 865899.0536 | 996206.1253 | 1061266.751 |
| Com_5765_neg | (S)-beta-Aminoisobutyric Acid | C4 H9 N O2 | 103.06335 | 2.669 | 1447605.207 | 1068042.619 | 1122110.655 | 1302032.089 | 1457727.522 | 1024446.433 | 1273533.517 | 1226420.158 | 1213075.569 |
| Com_5839_neg | 2-Amino-3-(4-hydroxy-3-methoxyphenyl)propanoic acid | C10 H13 N O4 | 211.08455 | 3.82 | 1023623.347 | 761806.989 | 1454252.487 | 662900.0564 | 568607.5838 | 488225.3229 | 624174.5197 | 867303.9468 | 805773.9008 |
| Com_5856_neg | LPC 22:0 | C30 H62 N O7 P | 625.43165 | 11.843 | 1141112.537 | 1388287.418 | 683741.6608 | 760055.8189 | 1648190.553 | 1279362.448 | 1449237.209 | 1224827.993 | 1379657.119 |
| Com_5903_neg | dAMP | C10 H14 N5 O6 P | 331.06682 | 4.9 | 1373649.003 | 315619.8856 | 1130259.613 | 857618.7946 | 504632.2167 | 1082681.394 | 776282.1941 | 1023271.837 | 1109065.985 |
| Com_5985_neg | trans-2-Methyl-2-pentenoic Acid | C6 H10 O2 | 114.06814 | 5.604 | 1100014.721 | 678040.5991 | 850567.1035 | 915254.7712 | 792182.3953 | 1325727.257 | 1234989.401 | 1427826.978 | 1022520.121 |
| Com_6144_neg | 1-(4-methylphenyl)-3-(2-pyridylthio)pyrrolidine-2,5-dione | C16 H14 N2 O2 S | 299.07717 | 3.849 | 1010895.445 | 1254326.866 | 1044214.516 | 573259.9505 | 1211816.417 | 562449.1136 | 1001455.378 | 975910.9525 | 974794.826 |
| Com_6177_neg | Indoxylsulfuric acid | C8 H7 N O4 S | 213.00962 | 0.532 | 941572.0504 | 1234476.894 | 826378.2175 | 837511.5042 | 1340575.878 | 1281109.526 | 1037644.808 | 849951.5263 | 670463.8802 |
| Com_6237_neg | Cer-EODS (d22:0/12:0-O-18:2) | C52 H99 N O5 | 817.75386 | 10.204 | 1167515.54 | 663703.4564 | 760750.2762 | 1084892.048 | 933620.7023 | 1253163.923 | 889830.5409 | 1121345.665 | 772756.3162 |
| Com_6373_neg | LysoPC 18:0 | C26 H54 N O7 P | 523.36464 | 10.614 | 784231.7502 | 621856.6205 | 596657.0857 | 590572.9428 | 592436.1431 | 568610.5732 | 1126826.657 | 851534.8188 | 741372.1388 |
| Com_6443_neg | o-Cresol | C7 H8 O | 108.05762 | 0.535 | 1043423.523 | 1040785.103 | 976961.5657 | 1181729.372 | 918322.8776 | 1076580.388 | 1002310.535 | 962279.8029 | 629111.7249 |
| Com_6690_neg | D-Ala-D-Ala | C6 H12 N2 O3 | 160.08466 | 1.398 | 674833.2695 | 787329.9386 | 842612.6587 | 907534.9729 | 851221.8703 | 863588.3336 | 897716.3942 | 770408.5438 | 1010908.309 |
| Com_6777_neg | 3,8,9-trihydroxy-10-propyl-3,4,5,8,9,10-hexahydro-2H-oxecin-2-one | C12 H20 O5 | 290.13804 | 3.844 | 279623.5085 | 509431.5686 | 864409.3404 | 377643.5344 | 560334.4717 | 615650.3226 | 202142.1488 | 276777.2346 | 365620.2053 |
| Com_7026_neg | 5-S-cysteinyldopaquinone | C12 H14 N2 O6 S | 314.05594 | 5.329 | 425453.6944 | 582267.4644 | 251209.9026 | 313717.0379 | 821179.0774 | 437851.7463 | 374726.788 | 255466.3434 | 385247.1554 |
| Com_7318_neg | PE (18:0/20:5) | C43 H76 N O8 P | 765.5345 | 11.655 | 243938.6571 | 235083.9903 | 285107.8897 | 257864.7875 | 543032.0754 | 217640.4752 | 281536.7266 | 437293.1944 | 221501.0188 |
| Com_7542_neg | Deoxyinosine | C10 H12 N4 O4 | 252.08603 | 3.834 | 19510.59412 | 91435.60978 | 8127.082151 | 11339.21334 | 22352.50747 | 28218.04106 | 30650.78438 | 29300.8552 | 20890.76921 |
| Com_5_neg | Glycocholic acid | C26 H43 N O6 | 465.30905 | 7.074 | 5908738210 | 6446793652 | 6981244459 | 7988717941 | 1889809745 | 12214154451 | 7026682857 | 6694230086 | 6567378043 |
| Com_40_neg | Glycoursodeoxycholic acid | C26 H43 N O5 | 449.31421 | 7.56 | 995783813.1 | 902866460.7 | 773788730.6 | 1303793998 | 194079644.9 | 1540944797 | 1186788921 | 904779809.8 | 1105690758 |
| Com_32_neg | LPE 18:2 | C23 H44 N O7 P | 477.28563 | 9.091 | 1387409426 | 1539654588 | 1489378604 | 1491973284 | 1477341039 | 1385088513 | 1683034859 | 1555577306 | 1477314362 |
| Com_1208_neg | Tetradecanedioic acid | C14 H26 O4 | 258.18297 | 6.813 | 13292678.49 | 7555690.386 | 11594033.09 | 17947933.6 | 9737450.2 | 23452519.86 | 11770583.47 | 13986852.82 | 11914957.83 |
| Com_221_neg | (+/-)12(13)-DiHOME | C18 H34 O4 | 296.23505 | 8.084 | 105650167.3 | 83630031.66 | 115341263.3 | 165616950.3 | 84282115.75 | 154048745.1 | 148662343.8 | 120674633.8 | 130044177.7 |
| Com_83_neg | Inosine | C10 H12 N4 O5 | 268.08076 | 3.34 | 72638602.45 | 481959729.1 | 757624.6496 | 75568694.59 | 118057055.4 | 180559014.4 | 145784667.7 | 147190884.5 | 159181213 |
| Com_248_neg | Glycolithocholic acid | C26 H43 N O4 | 433.31925 | 8.278 | 87410137.12 | 68568441.56 | 129627925.6 | 90766264.45 | 28574621.71 | 87472851.59 | 93016981.92 | 88434766.46 | 82675233.08 |
| Com_346_neg | LPE 18:3 | C23 H42 N O7 P | 475.26999 | 8.637 | 69817258.58 | 81745851.94 | 79158139.21 | 61838235.59 | 50044439.19 | 55647390.9 | 86079769.37 | 77991100.25 | 69993518.3 |
| Com_255_neg | LPE 18:1 | C23 H46 N O7 P | 479.30135 | 9.588 | 91775982.18 | 101993870.4 | 122583502.6 | 131940636.7 | 82782565.1 | 94880383.77 | 127259904.8 | 122015257.4 | 121016500.4 |
| Com_250_neg | LPE 20:3 | C25 H46 N O7 P | 503.30158 | 9.383 | 82424291.41 | 127437872.1 | 122299214.6 | 65586404.15 | 65085411 | 60404406.18 | 123084259.3 | 105925219.6 | 93100645.86 |
| Com_926_neg | Bilirubin | C33 H36 N4 O6 | 584.26403 | 10.827 | 12648657.92 | 26222456.08 | 12276682.59 | 22125252.19 | 13176999.51 | 10425288.03 | 20370446.1 | 13747203.34 | 11726142.89 |
| Com_391_neg | Delta-Tridecalactone | C13 H24 O2 | 212.17763 | 8.64 | 71221659.97 | 50213395.84 | 53441713.29 | 75192388.52 | 79027015.09 | 60727577.73 | 62418489.56 | 65372741.6 | 60622233.59 |
| Com_125_neg | dopaquinone | C9 H9 N O4 | 195.05294 | 5.578 | 274896791.6 | 150735620.8 | 177375081.6 | 204617031.7 | 79484215.57 | 169562119.9 | 162976431.2 | 169803124.1 | 177289716.9 |
| Com_696_neg | LPE 20:5 | C25 H42 N O7 P | 499.27037 | 8.593 | 23888805.56 | 36563528.32 | 31817468.69 | 24467730.23 | 17784556.66 | 28863828.59 | 36072493.07 | 33208184.18 | 27678457.49 |
| Com_212_neg | LPE 18:0 | C23 H48 N O7 P | 481.31699 | 10.159 | 122124321.2 | 130236969.4 | 143567474.2 | 158518069.1 | 133732001.4 | 138383395.4 | 141840925.2 | 159500729.9 | 135549166.1 |
| Com_596_neg | 9-HpOTrE | C18 H30 O4 | 310.2144 | 7.969 | 29396066.23 | 37144191.9 | 43762008.53 | 43850884.31 | 38218007.26 | 45025779.42 | 35427081.79 | 34329888.87 | 32964033.59 |
| Com_51_neg | Arachidonic acid | C20 H32 O2 | 304.24014 | 9.946 | 578985545.5 | 863836428.5 | 740499366.2 | 637363761.3 | 689122343.2 | 610730787.4 | 906630414.5 | 698962610.5 | 719568101.3 |
| Com_750_neg | cis-7-Hexadecenoic Acid | C16 H30 O2 | 254.2247 | 8.32 | 20267943.71 | 31028442.81 | 30628953.59 | 29294086.74 | 25183734.54 | 39461475.12 | 34131958.35 | 26040241.32 | 28077083.71 |
| Com_930_neg | LPE 22:4 | C27 H48 N O7 P | 529.31736 | 9.654 | 10677005.12 | 14019912.95 | 13553059.9 | 29613232.32 | 8739842.167 | 10048027.21 | 19118101.87 | 16985772.71 | 17575561.44 |
| Com_4969_neg | N-Acetylsphingosine | C20 H39 N O3 | 341.29281 | 10.51 | 1746088.196 | 1921924.173 | 1385756.702 | 2145316.173 | 1513041.467 | 2218323.214 | 2175942.052 | 2084997.75 | 2196214.565 |
| Com_1514_neg | LPE 15:0 | C20 H42 N O7 P | 439.27011 | 8.868 | 14041211.36 | 10859626.52 | 11406325.39 | 14888348.35 | 9680314.174 | 9115224.475 | 11945834.93 | 9957440.198 | 13433339.22 |
| Com_140_neg | LPE 20:4 | C25 H44 N O7 P | 465.30944 | 9.026 | 152495958.5 | 243931988.1 | 169804155.1 | 226144023.1 | 139752540.9 | 116551999.7 | 225198810.1 | 178828014.2 | 199292313.9 |
| Com_3669_neg | N-Oleoyl Glycine | C20 H37 N O3 | 339.27732 | 9.901 | 2931941.254 | 1886898.98 | 3224636.803 | 2704159.217 | 1937935.782 | 4247877.634 | 3917852.796 | 3740041.103 | 2878791.12 |
| Com_1763_neg | 6-Keto-prostaglandin f1alpha | C20 H34 O6 | 370.23534 | 6.029 | 8170814.783 | 8195762.911 | 7084396.399 | 9285761.144 | 14199913.22 | 3990080.828 | 8154213.207 | 7989428.282 | 9894203.171 |
| Com_790_neg | 1-Palmitoyl-Sn-Glycero-3-Phosphocholine | C24 H50 N O7 P | 495.333 | 9.837 | 20793574.25 | 22749982.37 | 24040508 | 22186026.45 | 23749224.37 | 24138157.5 | 32087450.37 | 23496534.86 | 22435986.14 |
| Com_3451_neg | Pregnenolone | C21 H32 O2 | 316.24178 | 9.663 | 3583593.143 | 3724878.072 | 3483207.319 | 3773156.627 | 3364013.026 | 3333821.112 | 4366166.977 | 3935199.986 | 4288499.888 |
| Com_3101_neg | 12,13-EODE | C18 H32 O3 | 296.23535 | 7.641 | 3495373.193 | 4434510.795 | 4437113.87 | 5839939.968 | 5449217.093 | 6559649.695 | 4437066.85 | 4750375.308 | 5560877.196 |
| Com_95_neg | 7-Ketodeoxycholic acid | C24 H38 O5 | 406.27199 | 6.804 | 406883197.6 | 282810952.8 | 152243568 | 291455687.9 | 250851816.5 | 515579518.8 | 329639887.7 | 294254073.3 | 299156910.4 |
| Com_5228_neg | P-Aminohippuric Acid | C9 H10 N2 O3 | 194.06915 | 4.982 | 1823493.29 | 1303910.177 | 1360809.849 | 2164200.958 | 1760322.683 | 2094142.919 | 1643879.873 | 1773088.795 | 1997505.335 |
| Com_2271_neg | SM (d14:0/20:0) | C39 H81 N2 O6 P | 750.58967 | 11.188 | 5868437.603 | 5891127.474 | 7153780.856 | 3217752.232 | 5986423.084 | 10490267.19 | 7089189.847 | 4172627.852 | 6556222.479 |
| Com_2435_neg | 3-(3,4-Dihydroxyphenyl)-2-Methylalanine | C10 H13 N O4 | 211.08449 | 5.813 | 7124006.548 | 7043172.846 | 5827005.508 | 6471943.833 | 7602986.831 | 7818731.952 | 6421835.926 | 7950280.144 | 7094302.063 |
| Com_3511_neg | 8(R)-Hydroxy-(5Z,9E,11Z,14Z)-eicosatetraenoic acid | C20 H32 O3 | 320.23491 | 9.118 | 4058955.204 | 3445616.712 | 2064531.305 | 3804893.779 | 3202759.646 | 2628296.79 | 2323694.341 | 2762202.075 | 2564027.095 |
| Com_3967_neg | L-Tyrosinemethylester | C10 H13 N O3 | 195.08959 | 5.906 | 3307349.317 | 2842216.818 | 1454775.378 | 2121450.4 | 1996322.664 | 2893465.235 | 2071573.429 | 2509030.5 | 2094125.807 |
| Com_280_neg | O-Acetyl-L-carnitine | C9 H17 N O4 | 203.11573 | 3.407 | 47158873.78 | 108050387.9 | 38164890.78 | 86114383.56 | 93891306.44 | 65097698.7 | 64534686.4 | 80879599.46 | 61747257.59 |
| Com_2653_neg | Lysope 14:0 | C19 H40 N O7 P | 425.25433 | 7.808 | 3758549.147 | 2734166.845 | 3919707.089 | 3549022.663 | 3341968.022 | 8365748.35 | 4954631.807 | 4832051.243 | 4295527.267 |
| Com_4271_neg | MAG (18:4) | C21 H34 O4 | 350.24582 | 8.445 | 2382979.783 | 2688727.349 | 1966841.372 | 3347937.844 | 2399691.285 | 3104518.74 | 2715677.756 | 2732243.963 | 2290007.881 |
| Com_4408_neg | H-Gly-Pro-OH | C7 H12 N2 O3 | 172.08471 | 1.38 | 1402599.09 | 1596457.175 | 1450787.144 | 931977.2979 | 3294745.826 | 2777795.009 | 1414963.034 | 1068525.536 | 1352516.18 |
| Com_736_neg | N-lactoyl-phenylalanine | C12 H15 N O4 | 237.1001 | 4.928 | 23995563.67 | 32037998.99 | 18789364.65 | 39543824.49 | 39287421.38 | 36104061.81 | 29292389.14 | 29462723.5 | 30336592.16 |
| Com_5662_neg | Lysops 22:5 | C28 H46 N O9 P | 571.28888 | 9.35 | 1223175.266 | 1211735.439 | 1577680.815 | 964287.0486 | 749996.9634 | 941413.4328 | 1130273.228 | 933663.7312 | 1119522.829 |

| Supplementary Table S5 HMDB classification note. | | | |
| --- | --- | --- | --- |
| SuperClass | Metabolites numbers | Metabolites | Metabolite_Descriptions |
| Alkaloids and derivatives | 1 | Com_787_neg | Reserpine |
| Benzenoids | 21 | Com_68_neg,Com_82_neg,Com_208_neg,Com_341_neg,Com_354_neg,Com_501_neg,Com_10_neg,Com_28_neg,Com_614_neg,Com_647_neg,Com_798_neg,Com_1207_neg,Com_1690_neg,Com_1844_neg,Com_1990_neg,Com_2355_neg,Com_2392_neg,Com_4150_neg,Com_4348_neg,Com_6443_neg,Com_5228_neg | Salicylic acid,Catechol,4-Methylcatechol,2,4-Dihydroxybenzoic acid,4-Nitrophenol,2-Hydroxyhippuric acid,Hippuric acid,4-Methylphenol,Phenylpyruvic acid,5-Sulfosalicylic acid,Butylparaben,2,5-Dimethylphenol,Paracetamol,Epinephrine,o-Toluic Acid,2-(3,4-dimethoxyphenyl)ethanamine,Pyrogallol,Bisphenol A,Homogentisic acid,o-Cresol,P-Aminohippuric Acid |
| Lipids and lipid-like molecules | 73 | Com_6_neg,Com_113_neg,Com_332_neg,Com_1076_neg,Com_1647_neg,Com_1812_neg,Com_1059_neg,Com_1127_neg,Com_20_neg,Com_24_neg,Com_37_neg,Com_58_neg,Com_74_neg,Com_89_neg,Com_92_neg,Com_109_neg,Com_134_neg,Com_176_neg,Com_268_neg,Com_295_neg,Com_297_neg,Com_299_neg,Com_340_neg,Com_353_neg,Com_492_neg,Com_498_neg,Com_517_neg,Com_520_neg,Com_592_neg,Com_603_neg,Com_612_neg,Com_661_neg,Com_662_neg,Com_796_neg,Com_805_neg,Com_858_neg,Com_1024_neg,Com_1145_neg,Com_1395_neg,Com_1413_neg,Com_1446_neg,Com_1453_neg,Com_1515_neg,Com_1643_neg,Com_1758_neg,Com_1915_neg,Com_2016_neg,Com_2147_neg,Com_2185_neg,Com_2228_neg,Com_2340_neg,Com_2610_neg,Com_2701_neg,Com_2749_neg,Com_2900_neg,Com_3402_neg,Com_4404_neg,Com_4590_neg,Com_4991_neg,Com_5033_neg,Com_5203_neg,Com_5405_neg,Com_5526_neg,Com_5_neg,Com_40_neg,Com_1208_neg,Com_248_neg,Com_51_neg,Com_1763_neg,Com_790_neg,Com_3451_neg,Com_95_neg,Com_280_neg | Cholic acid,Dodecanedioic acid,Adrenic acid,3-Methyladipic acid,Ethylmalonic acid,2-Isopropylmalic acid,Thromboxane B2,Docosahexaenoic Acid,LPC 18:1,Stearic acid,Elaidic acid,Deoxycholic acid,Dihydroroseoside,Azelaic acid,Taurochenodeoxycholic acid,2-Hydroxy-2-methylbutanoic acid,8Z,11Z,14Z-Eicosatrienoic acid,Arachidic acid,16-Hydroxyhexadecanoic acid,20-Hydroxy-(5Z,8Z,11Z,14Z)-eicosatetraenoic acid,Docosapentaenoic acid,Aldosterone,cis-5,8,11,14,17-Eicosapentaenoic acid,Chenodeoxycholic Acid,Octanedioic acid,Valproic acid,3-Methyl-2-oxobutanoic acid,Heptanoic acid,Lauric acid ethyl ester,Prostaglandin H2,Prostaglandin D2,2-Hydroxyisocaproic Acid,Hexadecanedioic acid,11(Z)-Eicosenoic acid,Prostaglandin D1,Lauric Acid,Docosanoic Acid,Erucic acid,Lignoceric acid,Citraconic acid,Glycerol 1-hexadecanoate,2-Methylpentanedioic acid,Prostaglandin B2,Prostaglandin H1,Nonadecanoic acid,Docosatrienoic acid,2-Arachidonyl glycerol ether,PC(16:0/16:0),trans-2-Butene-1,4-dicarboxylic Acid,Pentadecanoic Acid,Mevalonic acid,4-Oxoretinol,10-Undecenoic acid,Allolithocholic acid,Nervonic acid,2,3-Dinor-TXB2,Heneicosanoic acid,Calcitriol,Hydrocortisone acetate,4-Methylvaleric Acid,Ursolic acid,Capric acid,Leukotriene C4,Glycocholic acid,Glycoursodeoxycholic acid,Tetradecanedioic acid,Glycolithocholic acid,Arachidonic acid,6-Keto-prostaglandin f1alpha,1-Palmitoyl-Sn-Glycero-3-Phosphocholine,Pregnenolone,7-Ketodeoxycholic acid,O-Acetyl-L-carnitine |
| Nucleosides, nucleotides, and analogues | 10 | Com_111_neg,Com_333_neg,Com_415_neg,Com_4083_neg,Com_537_neg,Com_3515_neg,Com_4450_neg,Com_5903_neg,Com_7542_neg,Com_83_neg | Uridine,2'-Deoxyuridine,Thymidine,Xanthosine,Guanosine,N4-Acetylcytidine,APS,dAMP,Deoxyinosine,Inosine |
| Organic acids and derivatives | 44 | Com_110_neg,Com_138_neg,Com_203_neg,Com_219_neg,Com_267_neg,Com_336_neg,Com_439_neg,Com_446_neg,Com_532_neg,Com_587_neg,Com_2110_neg,Com_3165_neg,Com_18_neg,Com_502_neg,Com_1474_neg,Com_3130_neg,Com_35_neg,Com_409_neg,Com_529_neg,Com_597_neg,Com_683_neg,Com_722_neg,Com_831_neg,Com_854_neg,Com_1020_neg,Com_1303_neg,Com_1411_neg,Com_1591_neg,Com_1674_neg,Com_1806_neg,Com_1824_neg,Com_1941_neg,Com_2040_neg,Com_2300_neg,Com_2710_neg,Com_3502_neg,Com_4173_neg,Com_5177_neg,Com_5694_neg,Com_5765_neg,Com_6177_neg,Com_6690_neg,Com_125_neg,Com_736_neg | N-Isovalerylglycine,L-Phenylalanine,3-Hydroxybutyric acid,3-Indoxyl sulphate,Citric acid,3-Hydroxy-L-proline,N-Tigloylglycine,5-Aminovaleric acid,N-Acetylornithine,L-Histidine,N-Acetylglycine,Taurine,Phenylacetylglycine,Glycyl-L-leucine,Carnosine,Levothyroxine,2-Ketohexanoic acid,Levodopa,2-Furoylglycine,Citrulline,N-Isobutyrylglycine,alpha-Ketoglutaric acid,N-Acetyl-L-leucine,Ornithine,Glutaric Acid,4-Acetamidobutyric Acid,N-Propionylglycine,Homoarginine,5-Aminopentanoate,cis-Aconitic acid,Methionine,trans-Aconitic acid,glutathione disulfide,Arginine,acetoacetate,N-(5-Aminopentyl)acetamide,Argininosuccinic acid,L-Methionine sulfone,N-Acetyl-L-methionine,(S)-beta-Aminoisobutyric Acid,Indoxylsulfuric acid,D-Ala-D-Ala,dopaquinone,N-lactoyl-phenylalanine |
| Organic nitrogen compounds | 1 | Com_2230_neg | Porphobilinogen |
| Organic oxygen compounds | 6 | Com_42_neg,Com_848_neg,Com_3442_neg,Com_1816_neg,Com_3083_neg,Com_3254_neg | D-(-)-Fructose,L-Threonic acid,Acetaminophen glucuronide,Kynurenine,Gluconolactone,3-Phosphoglyceric acid |
| Organoheterocyclic compounds | 17 | Com_30_neg,Com_1263_neg,Com_2739_neg,Com_75_neg,Com_123_neg,Com_126_neg,Com_372_neg,Com_431_neg,Com_651_neg,Com_801_neg,Com_2017_neg,Com_3432_neg,Com_3892_neg,Com_4143_neg,Com_4610_neg,Com_5685_neg,Com_926_neg | Uric acid,2-Furoic acid,4-Pyridoxic acid,DL-Tryptophan,Sulfaquinoxaline,5,6-dihydroxyindole,Allantoin,Indole-2-carboxylic acid,Ascorbic acid,L-Ascorbic acid 2-sulfate,L-Ascorbate,Kynurenic acid,Xanthine,5-Hydroxytryptophol,Purine,Pyridoxamine,Bilirubin |
| Phenylpropanoids and polyketides | 5 | Com_419_neg,Com_1018_neg,Com_1773_neg,Com_1827_neg,Com_2983_neg | 3-(4-Hydroxyphenyl)propionic acid,Xanthohumol,2-Phenylpropionic acid,trans-Cinnamic acid,P-Coumaric acid |
